# Seeing beyond the symptoms: biomarkers and brain regions linked to cognitive decline in Alzheimer’s disease

**DOI:** 10.3389/fnagi.2024.1356656

**Published:** 2024-05-15

**Authors:** Seyed Hani Hojjati, Abbas Babajani-Feremi, for the Alzheimer’s Disease Neuroimaging Initiative

**Affiliations:** ^1^Department of Radiology, Weill Cornell Medicine, Brain Health Imaging Institute, New York, NY, United States; ^2^Department of Neurology, University of Florida, Gainesville, FL, United States; ^3^Magnetoencephalography (MEG) Lab, The Norman Fixel Institute of Neurological Diseases, University of Florida Health, Gainesville, FL, United States

**Keywords:** Alzheimer’s disease (AD), biomarkers, neuroimaging, cognitive scores, early detection, ensemble regression tree (ERT), machine learning

## Abstract

**Objective:**

Early Alzheimer’s disease (AD) diagnosis remains challenging, necessitating specific biomarkers for timely detection. This study aimed to identify such biomarkers and explore their associations with cognitive decline.

**Methods:**

A cohort of 1759 individuals across cognitive aging stages, including healthy controls (HC), mild cognitive impairment (MCI), and AD, was examined. Utilizing nine biomarkers from structural MRI (sMRI), diffusion tensor imaging (DTI), and positron emission tomography (PET), predictions were made for Mini-Mental State Examination (MMSE), Clinical Dementia Rating Scale Sum of Boxes (CDRSB), and Alzheimer’s Disease Assessment Scale-Cognitive Subscale (ADAS). Biomarkers included four sMRI (e.g., average thickness [ATH]), four DTI (e.g., mean diffusivity [MD]), and one PET Amyloid-β (Aβ) measure. Ensemble regression tree (ERT) technique with bagging and random forest approaches were applied in four groups (HC/MCI, HC/AD, MCI/AD, and HC/MCI/AD).

**Results:**

Aβ emerged as a robust predictor of cognitive scores, particularly in late-stage AD. Volumetric measures, notably ATH, consistently correlated with cognitive scores across early and late disease stages. Additionally, ADAS demonstrated links to various neuroimaging biomarkers in all subject groups, highlighting its efficacy in monitoring brain changes throughout disease progression. ERT identified key brain regions associated with cognitive scores, such as the right transverse temporal region for Aβ, left and right entorhinal cortex, left inferior temporal gyrus, and left middle temporal gyrus for ATH, and the left uncinate fasciculus for MD.

**Conclusion:**

This study underscores the importance of an interdisciplinary approach in understanding AD mechanisms, offering potential contributions to early biomarker development.

## 1 Introduction

Alzheimer’s disease (AD) is a condition that gradually affects brain function over time. It is a complex disease that results in progressive pathological changes in the brain’s biochemical and biological processes, leading to permanent impairment of cognitive functions (Petersen et al., 1999; Petersen, 2004; Gomar et al., 2011). Neuropsychological assessments play a pivotal role in identifying AD symptoms across various cognitive domains, including memory, language, and executive function (Bayles, 1982; Moss et al., 1986; Welsh et al., 1991; Almkvist, 1996; Chen et al., 2000). However, early-stage AD diagnosis poses a significant challenge, as symptoms may not be fully manifest. Neuroimaging emerges as an invaluable tool for improving early diagnosis since changes in the brain occur years before symptomatic presentation (Weintraub et al., 2012; Sperling et al., 2014; Pontecorvo et al., 2017). Therefore, predicting neuropsychological assessments based on neuroimaging biomarkers in both early/asymptomatic and late/symptomatic stages of AD is critical for comprehending the initial symptoms and unraveling the interplay between various patterns of impairment in brain regions affected by the disease (Bondi et al., 1995; Schmidt et al., 1996; Zhou et al., 2013).

Neuroimaging proves highly effective in tracking AD progression and identifying sensitive indicators, especially in the early stages. Various neuroimaging tools, including structural magnetic resonance imaging (sMRI), positron emission tomography (PET), and diffusion tensor imaging (DTI), have been used to predict AD progression through neuropsychological assessment (Wen et al., 2019; Tabarestani et al., 2020). Recent studies have used volumetric biomarkers based on sMRI, including gray matter volume and cortical thickness, to investigate the relationship between neuropsychological scores and neuroimaging biomarkers (Frisoni et al., 2002; Apostolova et al., 2006; Frisoni et al., 2010; Zhou et al., 2013). Additionally, PET has allowed for a detailed investigation of associations between neuropsychological assessment and proteinopathies during AD pathogenesis. While markers like 18F-fluorodeoxyglucose (FDG), tau tangles, and amyloid-β (Aβ) plaques are key in AD pathology, previous studies have shown that Aβ deposition has a weak correlation with cognition decline (Hedden et al., 2013). In contrast, tau and FDG pathological changes have been reported as strong biomarkers that are associated with cognitive decline especially in the later stage due to atrophy (Koss et al., 2016; Huber et al., 2018; Iida et al., 2021). Neuropathological studies suggest that tau mediates the link between Aβ and cognitive decline, primarily manifesting in patients with mild cognitive impairment (MCI) and AD (Choi et al., 2018; Tabarestani et al., 2020). Moreover, the amyloid cascade hypothesis suggests that the accumulation of Aβ initiates a sequence of events leading to AD development (Hardy and Higgins, 1992; Reitz, 2012). This hypothesis suggests that Aβ buildup triggers inflammation and oxidative stress, damaging neurons and disrupting their communication, subsequently resulting in classic AD symptoms like memory loss, cognitive decline, and behavioral changes (Busche and Hyman, 2020; Koller et al., 2021). Recent studies have also harnessed DTI for detecting micro-structural changes that are typically invisible in anatomical scans and undetectable by PET (Wen et al., 2019; Becerra-Laparra et al., 2020). Despite the potential of DTI biomarkers to predict neuropsychological assessments, they remain underutilized. The evolving understanding of AD neurobiology suggests that it is a multifaceted and heterogeneous disease that cannot be explained by a single biomarker or modality alone (Jack et al., 2018). Therefore, multimodal imaging techniques are essential for exploring the complex and consistent changes that accompany AD.

The application of machine learning and regression analysis offers promise for early diagnosis and treatment of cognitive impairments, including AD. In recent years, various regression methods such as least squares, support vectors, lassos, and regression trees have been successfully used to predict neuropsychological scores based on neuroimaging biomarkers (Zhang et al., 2012; Moradi et al., 2017; Tabarestani et al., 2020). Machine learning, in particular, has emerged as a compelling technique for predicting cognitive scores (Fan et al., 2010; Duc et al., 2020; Tabarestani et al., 2020). Recent studies have also shown a strong interest in integrating features from different neuroimaging modalities to predict neuropsychological assessments based on machine learning techniques (Bhagwat et al., 2019; Tabarestani et al., 2020; Hojjati and Babajani-Feremi, 2022). Some studies have combined neuropsychological scores with neuroimaging biomarkers to find the progression trend or predict neuropsychological assessments (Gill et al., 2020; Tabarestani et al., 2020; Kuo et al., 2023). However, the use of several predictors in such studies can result in the inclusion of unrelated information in their different prediction tasks, leading to a decrease in regression performance (Zhou et al., 2012). Additionally, predicting one cognitive assessment based on another can introduce bias due to their high correlation (Tabarestani et al., 2020). In recent years, neuropsychological predictions have predominantly incorporated single-modal data or found integrative methods for combining data across multiple biomarkers from multimodal data. However, these methods have not sufficiently captured the heterogeneity of AD progression.

Prior research has primarily focused on achieving high accuracy in classifying subjects or minimizing errors in estimating cognitive scores through regression analysis. Yet, most studies that have used multimodal feature domains have paid little attention to the differences between feature domains in the sample data. Advancing clinical research and drug development necessitates greater emphasis on understanding the relationship between effective biomarkers and the brain regions impacted by AD throughout its stages of progression. Our Study aimed to bridge the gap between these research domains, providing significant value in the context of clinical and therapeutic investigations.

We conducted separate analyses of different modalities and biomarkers, exploring the progression of AD. We identified the most significant predictive biomarkers and brain regions for each cognitive assessment, utilizing three neuroimaging modalities (sMRI, DTI, and PET) to predict AD progression using the Mini-Mental State Examination (MMSE), Clinical Dementia Rating Sum of Boxes (CDRSB), and Alzheimer’s Disease Assessment Scale (ADAS). Leveraging a substantial sample set of 1759 individuals, covering a spectrum of normal aging, MCI, and AD, we extracted nine competing biomarkers. Using the ensemble regression tree (ERT) techniques, we predicted target cognitive scores for four different groups: healthy controls (HC)/MCI, HC/AD, MCI/AD, and HC/MCI/AD. Through a robust integration of large sample sets, three complementary neuroimaging modalities, and nine different biomarkers, we were able to determine the extent to which each biomarker contributes to predicting the cognitive scores. Our investigation delves into the spatial characteristics of neuroimaging biomarkers and their association with cognitive assessments, shedding light on AD progression, particularly in its early stages. By developing a reliable biomarker based on each neuroimaging modality, there is a potential to capture the heterogeneity in the clinical evaluation of at-risk individuals and accelerate preventive strategies in the early stages of cognitive decline.

## 2 Materials and methods

### 2.1 Participants

This study utilized sMRI, DTI, PET, and neuropsychological data from a total of 1759 participants. The data were obtained from the Alzheimer’s disease prediction of longitudinal evolution (TADPOLE) challenge^1^ (Marinescu et al., 2018), which was sourced from the Alzheimer’s Disease Neuroimaging Initiative (ADNI) database (adni.loni.usc.edu).^2^ The current study included 648 healthy controls (294 males), 699 individuals with MCI (411 males), and 412 individuals with AD (234 males). Table 1 presents the demographic and clinical characteristics of the participants. Unstable HC and MCI subjects with any conversions or revisions were excluded (Hojjati et al., 2018). It is important to note that subjects did not necessarily require all three neuroimaging modalities, as each modality had been used in separate prediction tasks.

**Table 1:** Demographic and clinical data.

		HC	MCI	AD	Statistics
sMRI	Number	265	274	227	–
	Male/Female	124/141	160/114	122/105	*p* < 0.02*, X*^2^ = 7.35
	Age, year (mean ± SD)	73.91 ± 5.57	73.48 ± 7.38	74.43 ± 7.97	*p* = 0.31, *F* = 1.14
	CDRSB score (mean ± SD)	0.04 ± 0.14	1.28 ± 0.74	4.48 ± 1.80	*p* < 0.0001, *F* = 1086.27
	MMSE score (mean ± SD)	29.06 ± 1.18	27.63 ± 1.89	23.08 ± 2.67	*p* < 0.0001, *F* = 604.83
	ADAS score (mean ± SD)	8.61 ± 4.06	15.01 ± 6.22	29.23 ± 8.20	*p* < 0.0001, *F* = 680.29
DTI	Number	74	78	44	–
	Male/Female	31/43	48/30	28/16	*p* < 0.02*, X*^2^ = 7.78
	Age, year (mean ± SD)	72.64 ± 5.12	72.24 ± 7.41	74.66 ± 8.73	*p* = 0.16, *F* = 1.79
	CDRSB score (mean ± SD)	0.06 ± 0.16	1.25 ± 0.72	4.60 ± 1.51	*p* < 0.0001, *F* = 400.69
	MMSE score (mean ± SD)	28.82 ± 1.48	28.14 ± 1.57	23.40 ± 1.96	*p* < 0.0001, *F* = 167.74
	ADAS score (mean ± SD)	8.14 ± 4.21	14.29 ± 4.96	29.75 ± 7.82	*p* < 0.0001, *F* = 216.57
PET	Number	309	347	141	–
	Male/Female	139/170	203/144	84/57	*p* < 0.0006, *X*^2^ = 14.58
	Age, year (mean ± SD)	73.15 ± 5.81	72.00 ± 7.58	74.13 ± 8.04	*p* < 0.006, *F* = 5.13
	CDRSB score (mean ± SD)	0.04 ± 0.15	1.31 ± 0.81	4.52 ± 1.68	*p* < 0.0001, *F* = 1228.54
	MMSE score (mean ± SD)	29.02 ± 1.24	28.16 ± 1.64	23.11 ± 2.05	*p* < 0.0001, *F* = 712.18
	ADAS score (mean ± SD)	8.51 ± 4.15	13.45 ± 5.53	30.80 ± 8.38	*p* < 0.0001, *F* = 1,208

CDRSB, clinical dementia rating scale sum of boxes; ADAS, Alzheimer’s disease assessment scale; MMSE, mini-mental state examination; HC, healthy control; MCI, mild cognitive impairment; AD, Alzheimer’s disease.

### 2.2 Cognitive assessments

The current study aimed to predict cognitive scores using imaging biomarkers. Three cognitive scores were used as targets for prediction: MMSE, CDRSB, and ADAS. Each score highlights a different aspect of an individual’s neuropsychological status, depending on their stage of AD progression. The MMSE test is scored on a scale of 0–30, with a higher score indicating better cognitive function. The CDRSB and ADAS are scored on scales ranging from 0 to 18 and 0 to 70, respectively, with higher scores indicating more severe dementia and lower scores indicating milder dementia.

### 2.3 Neuroimaging data

We used various biomarkers from neuroimaging data as predictors in our machine learning models. Nine different types of biomarkers were extracted from the three neuroimaging modalities: sMRI, DTI, and PET. The aim of selecting these biomarkers was to establish a robust machine learning framework based on a substantial sample size. All imaging data had undergone prior processing, and post-processed measurements for each biomarker were collected (Marinescu et al., 2018). The sMRI biomarkers were grouped into four categories: volume of gray matter (VGM) in 68 cortical brain regions, average thickness (ATH) in 68 cortical brain regions, surface area (SA) in 70 cortical and subcortical brain regions, and volume of white matter (VWM) in 45 brain regions. The DTI biomarkers consist of fractional anisotropy (FA), mean diffusivity (MD), radial diffusivity (RD), and longitudinal diffusivity (LD), within 57 brain regions. Additionally, we utilized 18F-florbetapir PET imaging to capture Aβ deposition in 109 cortical and subcortical brain regions (see Supplementary Table S1). It is important to note that the regions marked with a red font in Supplementary Table S1 are similarly repeated in VGM, ATH, and Aβ, and they are based on the Desikan-Killiany cortical parcellation (Desikan et al., 2006). The selection of regions was based on the criterion of availability without any missing data across all participants and biomarkers in each modality. This rigorous approach aimed to maintain data integrity and consistency throughout the analysis, ensuring that the results accurately reflected the information from all participants without any gaps or inconsistencies due to missing values. These diverse biomarkers collectively formed the foundation of our machine learning model.

For grouping our samples, we included multiple cohorts within each group: HC/MCI, HC/AD, MCI/AD, and HC/MCI/AD. The decision to consider multiple cohorts as single group was influenced by several factors. First, examining biomarker performance across all four groups allows for a more comprehensive assessment of the data. It provides insights into how biomarkers perform across various combinations of clinical conditions, which may offer valuable insights not captured by analyzing individual conditions separately. Second, in clinical practice, patients often present with mixed symptoms or may transition between different clinical conditions over time. By considering multiple condition categories simultaneously, the analysis better reflects the real-world complexity of neurodegenerative diseases and provides a more accurate representation of biomarker performance in clinical settings. Finally, understanding how biomarkers perform across different clinical conditions is crucial for clinical decision-making and treatment planning. By considering all relevant condition categories, the analysis provides more actionable insights for clinicians and researchers working in the field of neurodegenerative diseases.

### 2.4 Modeling ensemble regression tree

In this study, the ensemble regression tree (ERT) was used to predict cognitive scores based on neuroimaging biomarkers. To ensure generalization of our models, we used a permutation process, repeated 500 iterations, to divide subjects into training (90%) and test (10%) sets across four groups: HC/MCI, HC/AD, MCI/AD, and HC/MCI/AD.

To address the bias and variance effects, we leveraged ensemble algorithms that combined the predictions of multiple estimators (Rokach, 2005). Our approach combined two ensemble learning techniques: bagging tree (BT) and random forest (RF). Bagging is an ensemble algorithm that fits multiple models on different subsets of a training set and then combines the predictions from all models. The BT aggregation aimed to decrease decision tree variance by merging multiple weak learners into stronger ones (Dey, 2016). We accomplished this by training 50 learner decision trees, each utilizing randomly selected subsets of data from training samples. The RF is an extension of bagging that also randomly selects subsets of features used in each data sample. We grew trees with surrogate splits to compute the error function for each variable at each split point (Springer and Kegelmeyer, 2008). For each biomarker (e.g., ATH, MD, and Aβ) the importance of each feature was determined by summing the changes in node risk resulting from splits on each feature and then dividing by the number of branch nodes. Node risk is defined as the mean squared error weighted by the node probability. Since we used surrogate split, the feature importance was calculated by summing changes in node risk across all splits at each branch node, including surrogate splits. The change in node risk highlights the difference between the risk of the parent node and the combined risk of its two children.

The feature importance metrics were derived from the average values across 500 iterations. Moreover, all reported mean squared error (MSE) values were based on the test sets across 500 iterations. We used analysis of variance (ANOVA) test followed by *post hoc* Tukey’s honestly significant difference (HSD) test with a family-wise error rate (FWER) of 0.01 to compare MSE of the nine biomarkers. Statistical significance for the categorical variables was assessed using chi-squared (*χ*2) test. It is noteworthy that we investigated the significance of various biomarkers in predicting cognitive measures by comparing their performance under two aspects based on: (A) MSE of predicting the cognitive measures in Section 3.1; (B) Correlation coefficient between actual values and predicted values in Section 3.1; and (C) ranking the significant brain regions in Sections 3.2 and 3.3. Implementation of the ERT algorithm and statistical analyses were carried out using MATLAB (2019b, MathWorks, Natick, MA).

### 2.5 Calculation of normalized importance and ranking the significant brain regions

For calculating the normalized importance (NI) of each biomarker (e.g., ATH, MD, and Aβ), we started by averaging the feature importance values across 500 iterations of the ERT. Next, we computed the square root of the average feature importance matrix and normalized it across all features associated with that specific biomarker. Finally, we divided the normalized values by the average across features to standardize the NI values across biomarkers. This standardization step ensures that the variation of the NI is centered around one, facilitating clearer comparisons and interpretations across biomarkers. By employing this approach, we obtained NI values that effectively reflect the relative importance of each biomarker in predicting the target outcome, while also accommodating variations in feature importance and variability across different biomarkers.

To identify significant brain regions, we compared the NI values with those generated through a random permutation test. This involved randomly shuffling the actual cognitive scores 500 times, alongside 500 iterations of train and test separation. Subsequently, we determined the 95th percentile (calculated as the mean plus two standard deviations of the 500 permuted NI values) for each biomarker as a threshold. Brain regions with NI values surpassing this threshold were deemed to have a notable influence on predicting cognitive scores. It’s important to note that when plotting the NI for each biomarker and subject group, we arranged the indices so that the larger values of NI curves consistently descend from left to right. This trend suggests that regions with lower indices are the ones significantly contributing to outcomes.

To enhance clarity regarding our permutation test, we constructed Supplementary Figure S1 as an illustrative example. This figure displays NI values alongside permutation results for Aβ across 109 brain areas in predicting MMSE scores. The black plots represent the mean of the permutation results in each group, with the standard deviation also depicted. Significant features were identified based on the 95th percentile of the fitted normal distribution, roughly equivalent to the mean plus two standard deviations. In Supplementary Figure S1A, focusing on the HC/MCI group representing individuals with non to mild cognitive decline, our findings suggest that no statistically significant brain region exhibited an NI higher than the 95th percentile of the fitted normal distribution from the permutation test. Conversely, examining the AD cohort in HC/AD, MCI/AD, and HC/MCI/AD groups (Supplementary Figures S1B–D), we observed that NI values in several brain regions significantly surpassed the 95th percentile of the fitted normal distribution from the permutation test results. It’s important to note that for better visualization in, we only plotted the mean of the permutation results for the HC/MCI/AD groups. This simplification offers insight into the general pattern of permutations results, typically centered around 1.

Finally, to visualize the significant ranked brain regions, we standardized NI values across four groups of individuals (HC/MCI, HC/AD, MC/AD, and HC/MCI/AD) and color coded them from lowest NI value (dark blue) to highest NI value (dark red).

## 3 Results

### 3.1 Evaluating biomarker efficacy from normal cognition to severe dementia

**Figure 1:**
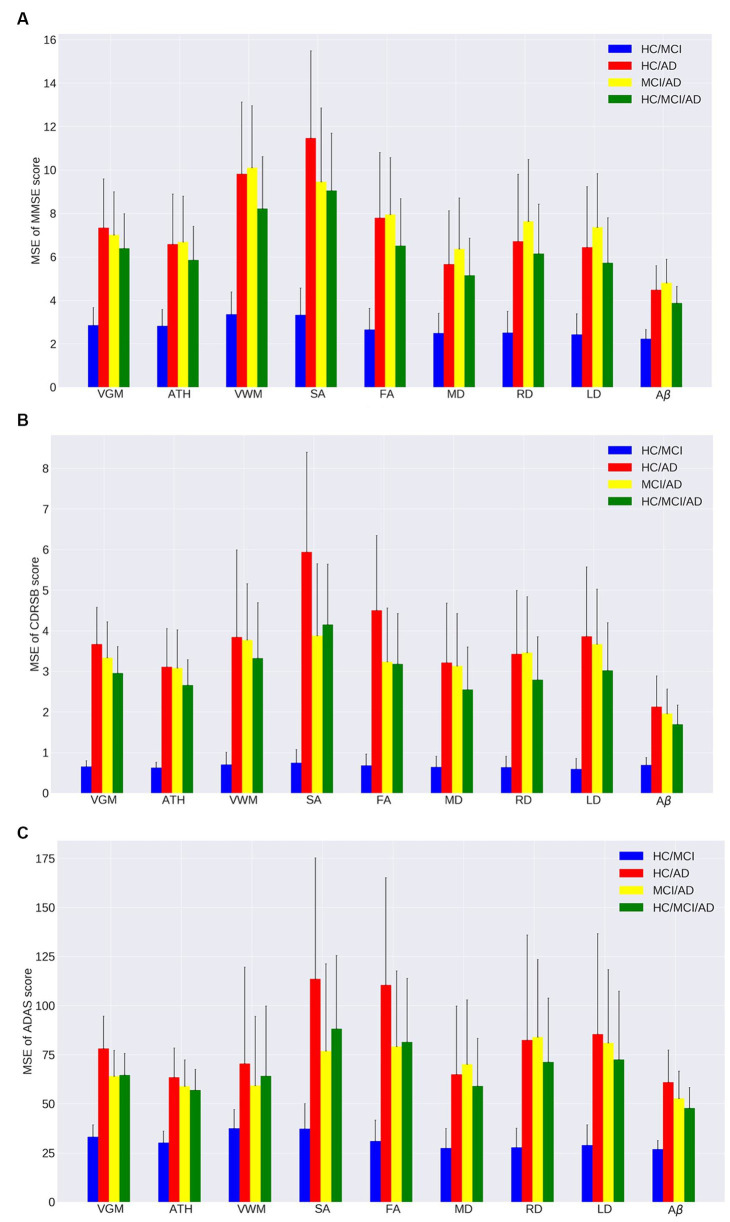
Mean squared error (MSE) of the ensemble regression tree models for predicting **(A)** MMSE, **(B)** CDRSB, and **(C)** ADAS based on nine neuroimaging biomarkers: VGM, ATH, VWM, SA, FA, MD, RD, LD, Aβ in four groups: HC/MCI, HC/AD, MCI/AD, and HC/MCI/AD.

**Figure 2:**
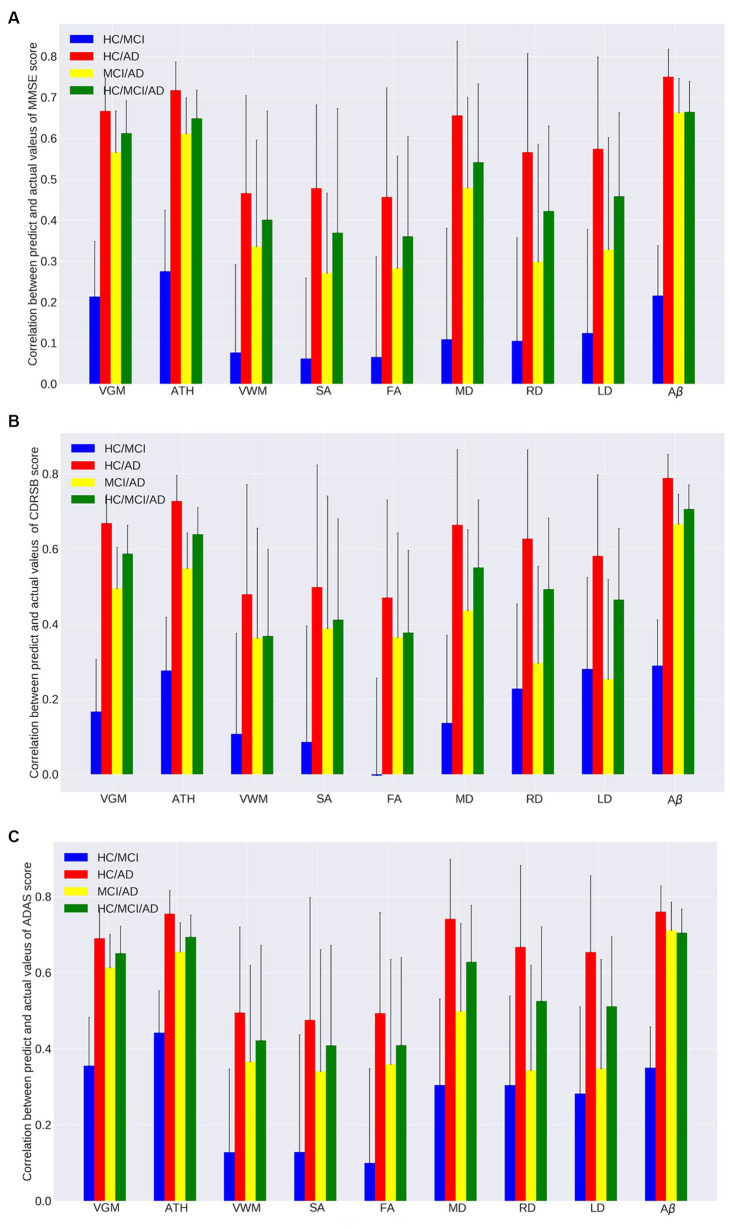
Correlation between actual values and predicted values of the ensemble regression tree models for predicting **(A)** MMSE, **(B)** CDRSB, and **(C)** ADAS based on nine neuroimaging biomarkers (VGM, ATH, VWM, SA, FA, MD, RD, LD, and Aβ) in four groups (HC/MCI, HC/AD, MCI/AD, and HC/MCI/AD).

Figures 1A–C, 2A–C and Tables 2, 3 provide insights into the performance of ERT models for predicting three cognitive scores (MMSE, CDRSB, and ADAS) based on nine neuroimaging biomarkers across four distinct groups (HC/MCI, HC/AD, MCI/AD, and HC/MCI/AD). Figures 1A–C and Table 2 offer results of MSE between predicted values and actual values, providing an indication of the predictive accuracy of the model. On the other hand, Figures 2A–C and Table 3 present data on the correlation coefficient between predicted values and actual values, showcasing the goodness of fit of the ERT model.

**Table 2:** MSE in three cognitive scores across nine biomarkers.

		HC/MCI	HC/AD	MCI/AD	HC/MCI/AD
MMSE	**VGM**	2.85 ± 0.82	7.34 ± 2.25	7.01 ± 1.99	6.39 ± 1.59
	**ATH**	2.82 ± 0.76	6.57 ± 2.32	6.68 ± 2.10	5.85 ± 1.55
	**VWM**	3.40 ± 1.08	9.93 ± 3.22	10.09 ± 2.79	8.06 ± 2.45
	**SA**	3.42 ± 1.21	11.50 ± 3.70	9.23 ± 3.18	9.01 ± 2.62
	**FA**	2.64 ± 0.98	7.79 ± 3.00	7.95 ± 2.62	6.51 ± 2.17
	**MD**	2.48 ± 0.91	5.66 ± 2.46	6.35 ± 2.36	5.147 ± 1.72
	**RD**	2.50 ± 0.99	6.71 ± 3.09	7.63 ± 2.84	6.15 ± 2.28
	**LD**	2.42 ± 0.96	6.44 ± 2.80	7.36 ± 2.47	5.72 ± 2.08
	**Aβ**	**2.22 ± 0.473**	**4.47 ± 1.12**	**4.80 ± 1.09**	**3.86 ± 0.77**
CDRSB	**VGM**	0.65 ± 0.14	3.66 ± 0.90	3.33 ± 0.89	2.95 ± 0.65
	**ATH**	0.62 ± 0.13	3.11 ± 0.94	3.08 ± 0.93	2.66 ± 0.62
	**VWM**	0.72 ± 0.32	3.94 ± 1.99	3.58 ± 1.47	3.21 ± 1.38
	**SA**	0.73 ± 0.35	5.96 ± 2.24	4.04 ± 1.75	4.38 ± 1.52
	**FA**	0.67 ± 0.28	4.50 ± 1.84	3.23 ± 1.32	3.18 ± 1.24
	**MD**	0.64 ± 0.27	3.21 ± 1.47	3.13 ± 1.29	2.54 ± 1.05
	**RD**	0.63 ± 0.27	3.42 ± 1.56	3.46 ± 1.38	2.79 ± 1.06
	**LD**	**0.59 ± 0.26**	3.85 ± 1.71	3.66 ± 1.36	3.02 ± 1.17
	**Aβ**	0.69 ± 0.18	**2.12 ± 0.76**	**1.96 ± 0.60**	**1.69 ± 0.47**
ADAS	**VGM**	33.15 ± 6.24	78.12 ± 16.64	64.07 ± 13.15	64.62 ± 11.13
	**ATH**	30.247 ± 5.94	63.54 ± 14.92	58.90 ± 13.47	56.97 ± 10.59
	**VWM**	37.80 ± 9.52	72.30 ± 48.57	58.53 ± 34.44	60.62 ± 3.84
	**SA**	36.79 ± 12.76	117.68 ± 64.02	74.70 ± 43.57	89.79 ± 39.34
	**FA**	31.07 ± 10.72	110.45 ± 54.71	79.16 ± 38.48	81.47 ± 32.41
	**MD**	27.52 ± 9.97	64.93 ± 34.88	70.09 ± 32.81	59.03 ± 24.38
	**RD**	27.79 ± 9.84	82.45 ± 583.56	83.98 ± 39.54	71.24 ± 32.66
	**LD**	28.90 ± 10.51	85.42 ± 51.31	80.97 ± 37.42	72.52 ± 34.86
	**Aβ**	**26.84 ± 4.49**	**60.94 ± 16.47**	**52.79 ± 13.95**	**47.83 ± 10.54**

The lowest average MSE values within each group are highlighted in bold font. CDRSB, clinical dementia rating scale sum of boxes; ADAS, Alzheimer’s disease assessment scale; VGM, volume of gray matter; VWM, volume of white matter; SA, surface area; FA, fractional anisotropy; MD, mean diffusivity; RD, radial diffusivity; LD, longitudinal diffusivity; Aβ, amyloid-β; ANOVA, analysis of variance; HC, healthy control; MCI, mild cognitive impairment; AD, Alzheimer’s disease.

**Table 3:** Correlation between predicted and actual values of three cognitive scores across nine biomarkers.

		HC/MCI	HC/AD	MCI/AD	HC/MCI/AD
MMSE	**VGM**	0.21 ± 0.14	0.67 ± 0.08	0.56 ± 0.10	0.61 ± 0.07
	**ATH**	**0.27 ± 0.15**	0.71 ± 0.07	0.61 ± 0.09	0.64 ± 0.06
	**VWM**	0.06 ± 0.23	0.45 ± 0.24	0.30 ± 0.23	0.39 ± 0.27
	**SA**	0.08 ± 0.18	0.46 ± 0.19	0.28 ± 0.20	0.34 ± 0.31
	**FA**	0.07 ± 0.24	0.45 ± 0.26	0.28 ± 0.27	0.36 ± 0.24
	**MD**	0.11 ± 0.27	0.65 ± 0.18	0.47 ± 0.22	0.54 ± 0.19
	**RD**	0.11 ± 0.25	0.56 ± 0.2	0.30 ± 0.28	0.42 ± 0.20
	**LD**	0.12 ± 0.25	0.57 ± 0.22	0.32 ± 0.27	0.45 ± 0.20
	**Aβ**	0.22 ± 0.12	**0.75 ± 0.07**	**0.66 ± 0.08**	**0.66 ± 0.07**
CDRSB	**VGM**	0.16 ± 0.14	0.66 ± 0.07	0.49 ± 0.10	0.58 ± 0.07
	**ATH**	0.27 ± 0.14	0.72 ± 0.06	0.54 ± 0.09	0.63 ± 0.07
	**VWM**	0.14 ± 0.29	0.45 ± 0.28	0.37 ± 0.30	0.37 ± 0.23
	**SA**	0.06 ± 0.28	0.48 ± 0.31	0.34 ± 0.36	0.40 ± 0.26
	**FA**	−0.003 ± 0.26	0.47 ± 0.26	0.36 ± 0.27	0.37 ± 0.21
	**MD**	0.13 ± 0.23	0.66 ± 0.20	0.43 ± 0.21	0.55 ± 0.18
	**RD**	0.22 ± 0.22	0.62 ± 0.23	0.29 ± 0.25	0.49 ± 0.19
	**LD**	0.28 ± 0.24	0.58 ± 0.21	0.25 ± 0.26	0.46 ± 0.18
	**Aβ**	**0.29 ± 0.12**	**0.79 ± 0.06**	**0.66 ± 0.08**	**0.70 ± 0.06**
ADAS	**VGM**	0.35 ± 0.12	0.69 ± 0.07	0.61 ± 0.08	0.65 ± 0.07
	**ATH**	**0.44 ± 0.11**	0.75 ± 0.06	0.65 ± 0.07	0.69 ± 0.05
	**VWM**	0.10 ± 0.22	0.50 ± 0.24	0.35 ± 0.24	0.40 ± 0.26
	**SA**	0.08 ± 0.29	0.49 ± 0.28	0.37 ± 0.31	0.40 ± 0.27
	**FA**	0.09 ± 0.24	0.49 ± 0.26	0.35 ± 0.27	0.41 ± 0.23
	**MD**	0.30 ± 0.22	0.74 ± 0.25	0.49 ± 0.23	0.62 ± 0.14
	**RD**	0.30 ± 0.23	0.66 ± 0.21	0.34 ± 0.27	0.52 ± 0.19
	**LD**	0.28 ± 0.22	0.65 ± 0.20	0.34 ± 0.28	0.51 ± 0.18
	**Aβ**	0.34 ± 0.10	**0.76 ± 0.06**	**0.71 ± 0.07**	**0.70 ± 0.06**

The highest average correlation values within each group are highlighted in bold font. CDRSB, clinical dementia rating scale sum of boxes; ADAS, Alzheimer’s disease assessment scale; VGM, volume of gray matter; VWM, volume of white matter; SA, surface area; FA, fractional anisotropy; MD, mean diffusivity; RD, radial diffusivity; LD, longitudinal diffusivity; Aβ, amyloid-β; ANOVA, analysis of variance; HC, healthy control; MCI, mild cognitive impairment; AD, Alzheimer’s disease.

As observed by the results presented in Figures 1A–C, 2A–C and Tables 2, 3, determining the most effective biomarker within each subject group requires a comprehensive evaluation. This evaluation entails not only analyzing the MSE, which quantifies the average squared difference between predicted and actual values but also considering the correlation between these predicted and actual values. It is essential to note that in certain predictive models, particularly noticeable within the HC/MCI group, the biomarker with the lowest MSE values may not consistently exhibit the highest correlation with the actual values. This observation emphasizes the complexity of the predictive model’s performance assessment. While achieving the lowest MSE suggests optimal performance in terms of error metrics, it does not guarantee that the predicted and actual values align perfectly or adhere closely to a linear assumption.

We conducted one-way ANOVA across nine biomarkers for each group, examining MSE and correlation values in Tables 2, 3. The analyses involved multiple ANOVA tests to evaluate biomarkers performances across three cognitive scores (MMSE, CDRSB, and ADAS) and four groups (HC/MCI, HC/AD, MCI/AD, and HC/MCI/AD). The results of the one-way ANOVA on MSE values (Table 2) demonstrated statistically significant differences among at least two biomarkers (*p* < 0.0001, *F* > 16.97) across all groups and cognitive scores. The results of the one-way ANOVA on correlation values (Table 3) also show the significant differences among at least two biomarkers (*p* < 0.0001, *F* > 92.20) across all groups and cognitive scores. This finding suggests the potential presence of specific biomarkers demonstrating superior performance compared to others.

An in-depth pairwise examination utilizing Tukey HSD tests (considering FWER of 0.01) on the nine biomarkers for MMSE cognitive score prediction (Figures 1A, 2A) highlighted Aβ’s exceptional predictive capabilities in the presence of an AD cohort. Aβ emerged as a notably superior predictor, yielding significantly smaller MSE values and larger correlation (average correlation >0.66) compared to the other eight biomarkers (*p*-value <0.0001). This finding is also consistent in Figures 1B, 2B, emphasizing Aβ’s superiority in predicting CDRSB scores, where MSE values were significantly smaller and larger correlation (average correlation >0.66) than those based on other biomarkers (p-value <0.0001). For ADAS score, based on both MSE and correlation (Figures 1C, 2C) only in the HC/MCI/AD group, Aβ significantly (*p* < 0.0001) outperformed all other biomarkers (average correlation >0.70) in predicting ADAS scores.

The average results of MSE and correlation for sMRI modality biomarkers across all cognitive scores, as shown in Tables 2, 3, consistently demonstrate that ATH outperforms the other three biomarkers (VGM, VWM, and SA) within this modality. Similarly, the assessment of DTI modality biomarkers across all cognitive scores indicates that MD surpasses the other three biomarkers (FA, RD, and LD) within the DTI modality across most groups and cognitive scores. These findings imply that ATH and MD exhibit superior predictive accuracy and stronger correlations with actual values across various cognitive scores compared to the other biomarkers in the sMRI and DTI modalities, respectively.

### 3.2 Identifying key biomarkers in AD progression

**Figure 3:**
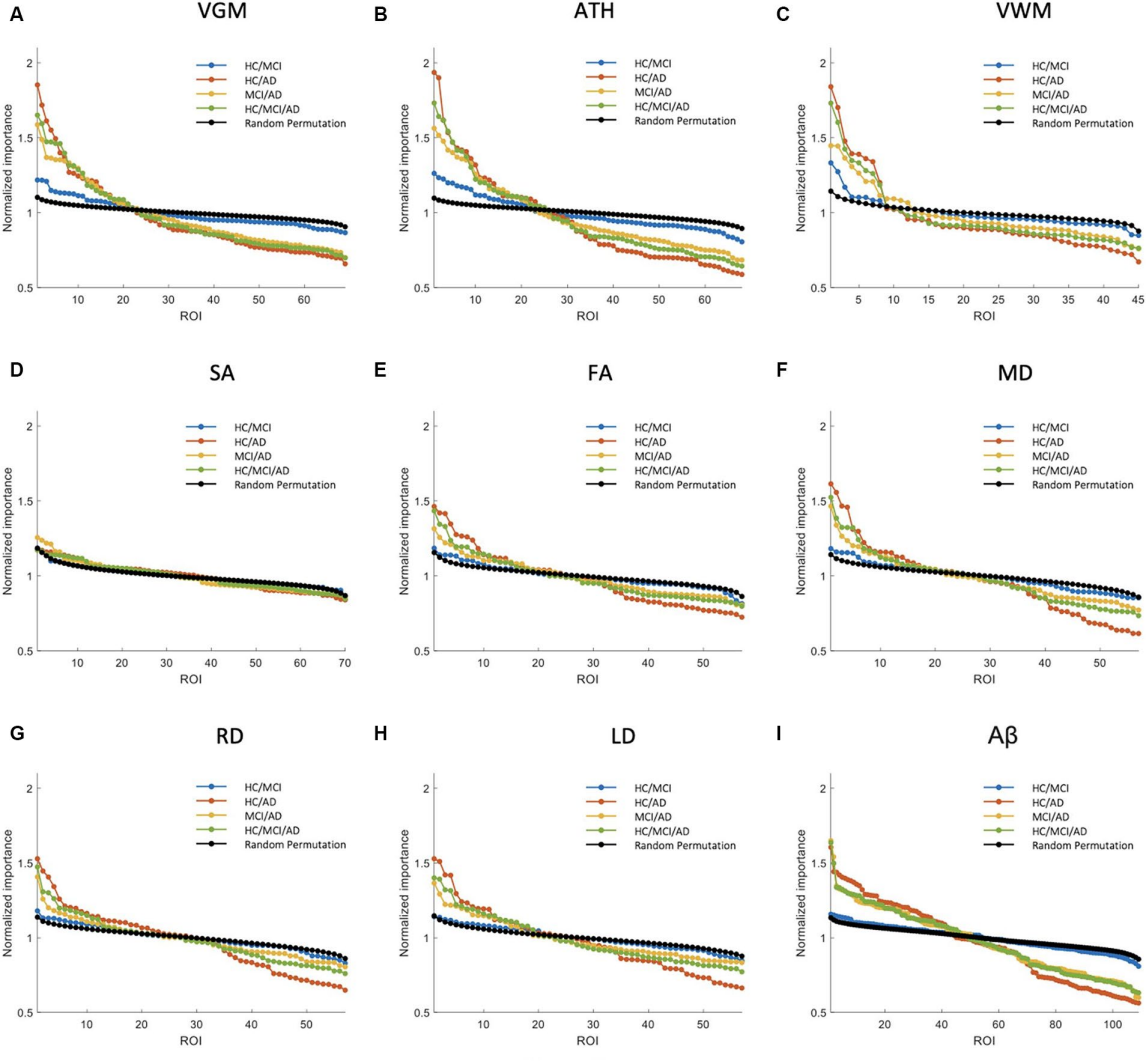
Feature importance for the prediction of MMSE score based on nine different biomarkers: **(A)** VGM, **(B)** ATH, **(C)** VWM, **(D)** SA, **(E)** FA, **(F)** MD, **(G)** RD, **(H)** LD, and **(I)** Aβ. The *x*-axis shows the brain regions considered in the prediction based on each biomarker. The *y*-axis represents the normalized importance value for four groups and a random permutation. Larger values of normalized importance in brain regions indicate a higher association between that region’s feature and the MMSE score.

**Figure 4:**
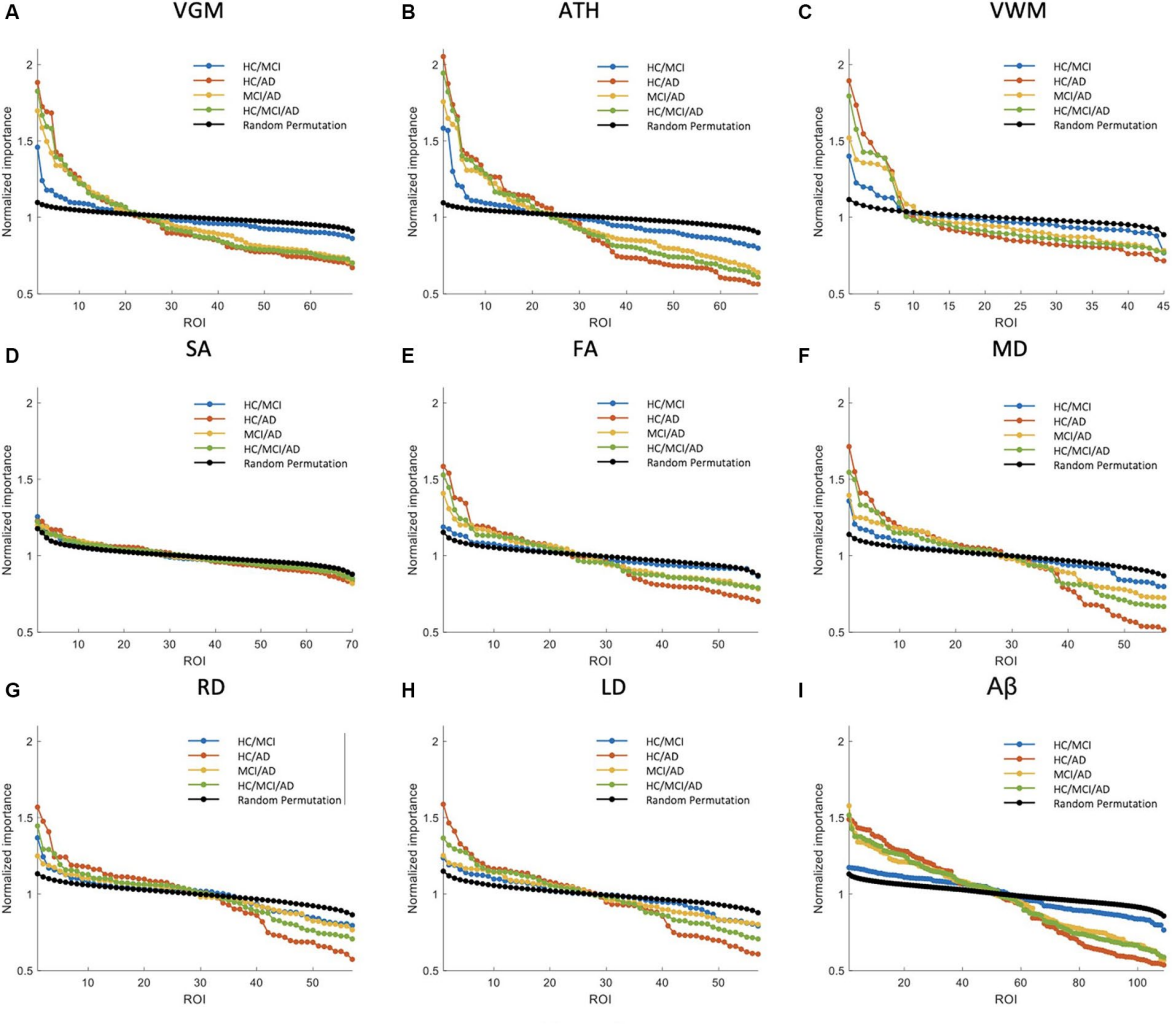
Feature importance for the prediction of ADAS score based on nine neuroimaging biomarkers: **(A)** VGM, **(B)** ATH, **(C)** VWM, **(D)** SA, **(E)** FA, **(F)** MD, **(G)** RD, **(H)** LD, and **(I)** Aβ. The *x*-axis shows the brain regions considered in the prediction based on each biomarker. The *y*-axis represents the normalized feature importance for four groups and a random permutation. Larger values of normalized importance in brain regions indicate a higher association between that region and the ADAS score.

In the previous section, we compared the biomarkers based on MSE values. In this section and Section 3.3, our focus is on comparing the biomarkers with the ranking of significant brain regions to identify the most effective biomarker. Figures 3, 4 primarily aim to identify the most crucial biomarkers in detecting the transition groups of subjects from normal cognition to severe cognitive decline. Each of the nine subplots in these figures corresponds to a neuroimaging biomarker, and their NI is compared across four groups of individuals (HC/MCI, HC/AD, MCI/AD, and HC/MCI/AD) in contrast to the average random permutation test results of HC/MCI/AD (only for visualization).

Figure 3 shows NI of the brain regions for predicting the MMSE score based on nine biomarkers. As shown in this figure, VGM, ATH, and VWM biomarkers demonstrated 3, 10, and 2 brain regions, respectively, with significantly larger NI in the HC/MCI group compared to random permutation test results. In contrast, we failed to identify any brain region with significantly larger NI in the HC/MCI group compared to random permutation based on Aβ. The presence of AD cohort in the other three groups increased the likelihood of identifying important brain regions. Specifically, as shown in Figure 3I, we found Aβ in more than 30 brain regions with NI significantly larger in groups with AD (HC/AD, MCI/AD, and HC/MCI/AD) than in the random permutation or HC/MCI group. Figures 3A–C, along with Figure 3I, illustrated that despite Aβ affecting more brain regions in the presence of AD cohort, the sMRI biomarkers VGM, VWM, and especially ATH achieved higher NI values. Figure 3D highlighted almost non-existent association between SA and MMSE scores. Additionally, DTI biomarkers (FA, MD, RD, and LD), indicated a significant difference in the NI of five brain regions between groups with and without AD.

Supplementary Figure S2 shows NI values for the prediction of CDRSB. The CDRSB prediction aligns closely with the MMSE score prediction, as shown in Figure 3, but the presence of the AD group led to higher NI in CDRSB compared to MMSE scores (Supplementary Figures S2A,B).

The NI results for ADAS prediction are illustrated in Figure 4. The HC/MCI group, as shown in this figure, provided particularly interesting results. Almost all biomarkers identified at least one significant brain region, not only in groups containing AD individuals but also in the HC/MCI group. Consequently, several brain regions were affected by all nine neuroimaging biomarkers. In the HC/MCI group, ATH emerged as the best predictor of ADAS scores, with two brain regions having NIs exceeding 1.5. Among DTI biomarkers, MD proved to be the most effective. In the MCI/AD, HC/AD, and HC/MCI/AD groups, which included the AD patients, 45, 25, and 15 brain regions, respectively, contributed to the prediction of Aβ, ATH, and MD.

### 3.3 Most discriminatory brain regions from normal cognition to severe dementia

**Figure 5:**
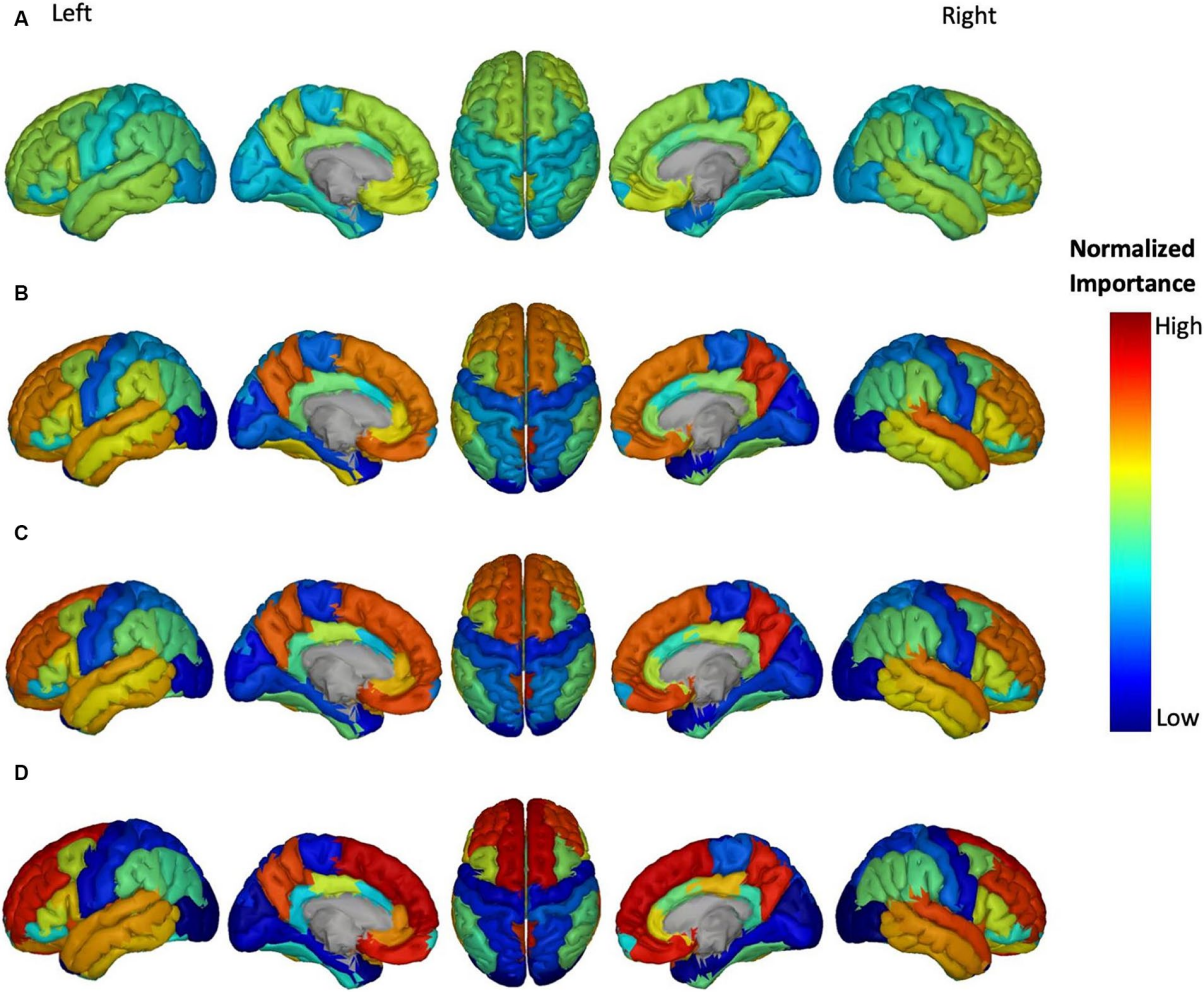
Feature importance of Aβ in brain regions for the prediction of ADAS score in four groups: **(A)** HC/MCI, **(B)** HC/AD, **(C)** MCI/AD, and **(D)** HC/MCI/AD. The groups of subjects show small to large differences in the cognitive scores from normal aging to AD, from top to bottom. The color map is based on the normalized feature importance values.

**Figure 6:**
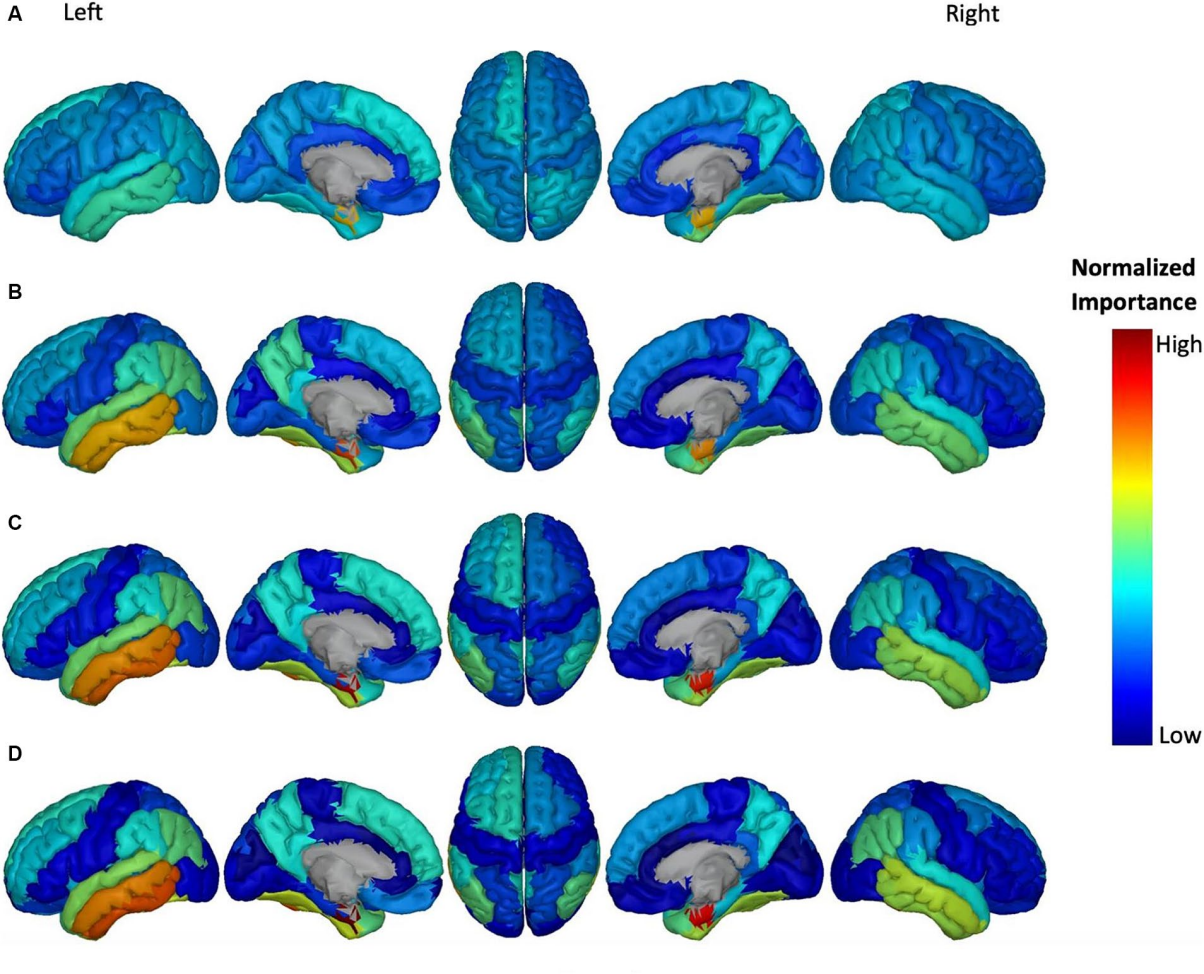
Feature importance of ATH in cortical brain regions for the prediction of ADAS score in four groups: **(A)** HC/MCI, **(B)** HC/AD, **(C)** MCI/AD, and **(D)** HC/MCI/AD. The groups of subjects show small to large differences in the cognitive scores from normal aging to AD, from top to bottom. The color map is based on the normalized feature importance values.

**Figure 7:**
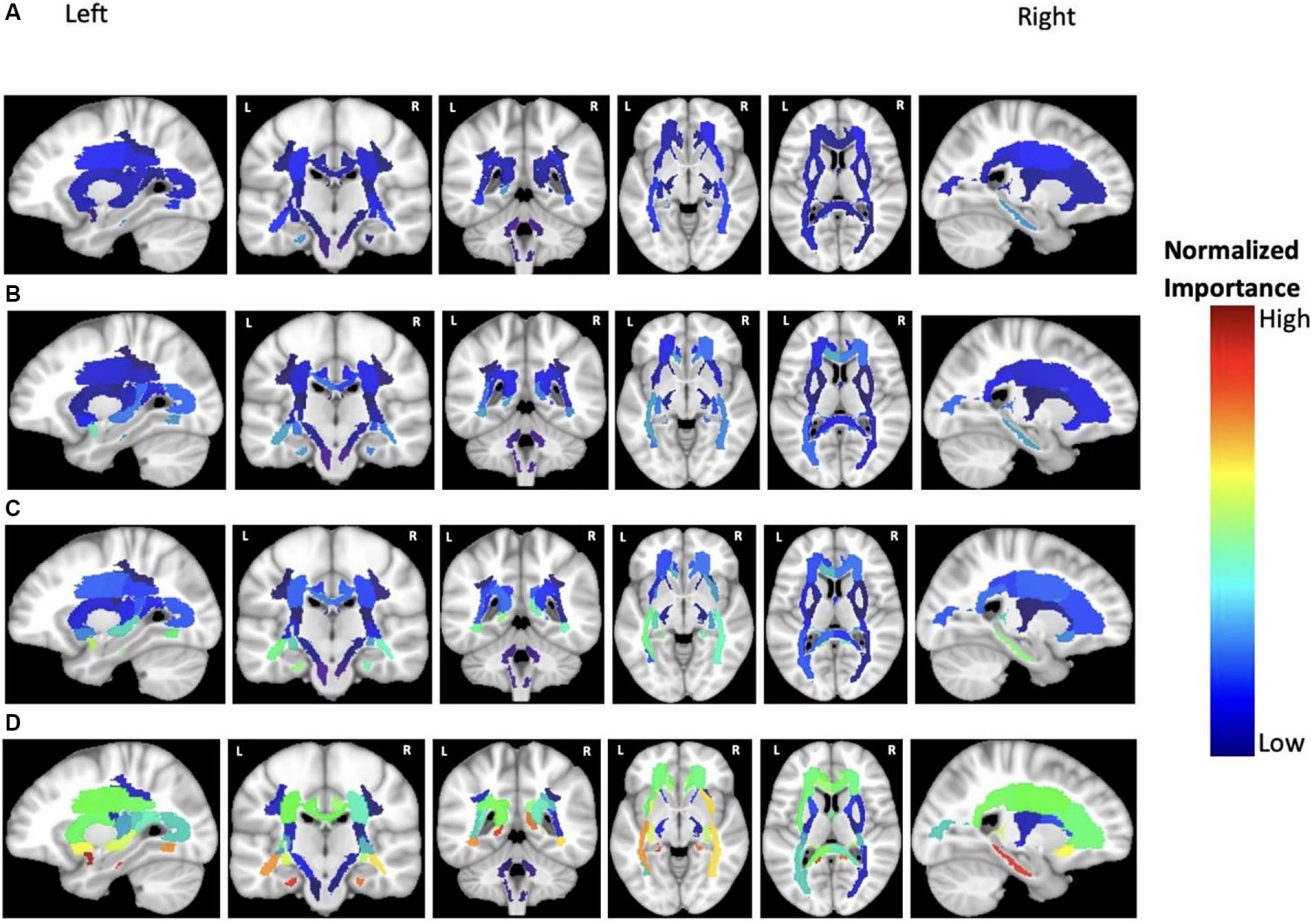
Feature importance of MD in cortical brain regions for the prediction of ADAS score in four groups: **(A)** HC/MCI, **(B)** HC/AD, **(C)** MCI/AD, and **(D)** HC/MCI/AD. The groups of subjects show small to large differences in the cognitive scores from normal aging to AD, from top to bottom. The color map is based on the normalized feature importance values.

Based on our prediction models, we observed several associations between ADAS and neuroimaging biomarkers across four groups of individuals transitioning from normal cognition to severe dementia. Aβ, ATH, and MD were identified as more important neuroimaging biomarkers for predicting cognitive scores based on PET, sMRI, and DTI, respectively. Figures 5–7 depict the NI values of these biomarkers for ADAS prediction. In the Supplementary materials, we also present the NI values of these three biomarkers for MMSE and CDRSB prediction (Supplementary Figures S3–S8). As previously mentioned, our analysis involved calculating feature importance across 68 regions based on the Desikan-Killiany cortical parcellation for both ATH and Aβ outcomes. Additionally, for Aβ, we examined 41 subcortical regions as detailed in Supplementary Table S1. However, Aβ in the subcortical regions did not demonstrate significant feature importance in comparison to the cortical regions, consistently ranking lower in importance. To ensure clarity and facilitate a more direct comparison between ATH and Aβ, we chose to focus exclusively on the 68 cortical regions common to both biomarkers in Figures 5, 6 and Supplementary Figures S3, S4, S6, S7.

The NI of cortical brain regions based on Aβ is illustrated in Figure 5. The top-ranked significant regions showed with dark yellow to red colors include the right and left transverse temporal gyrus, and right precuneus cortex in the MCI/AD group; right transverse temporal gyrus, right precuneus cortex in the HC/MCI/AD group; and right and left superior frontal gyrus, right and left transverse temporal gyrus, and right precuneus cortex in the HC/AD group. Notably, the right transverse temporal gyrus and right precuneus cortex consistently showed high NI throughout three groups with the presence of the AD group. The NI value of each brain region in the HC/MCI group was below the assumed threshold and not significant.

Figure 6 shows the most discriminatory brain regions according to ATH. The top-ranking significant regions showed with dark yellow to red colors for the HC/MCI group were the right and left entorhinal cortex and the right fusiform cortex. The other three groups (MCI/AD, HC/MCI/AD, and HC/AD) had the same top-ranking regions: left and right entorhinal cortex, left inferior temporal gyrus, and left middle temporal gyrus. In groups from one through four (1: HC/MCI, 2: MCI/AD, 3: HC/MCI/AD, and 4: HC/AD), the NI values of the left and right entorhinal cortex gradually increased, revealing the significant progression of these two regions based on ATH.

According to previous results and as shown in Figure 7, MD was the most discriminatory DTI biomarker. In each group, the top significant regions were as follows: left cingulum and right sagittal stratum in the HC/MCI group; left uncinate fasciculus in the MCI/AD group; left uncinate fasciculus and left cingulum in the HC/MCI/AD group; and left uncinate fasciculus and left cingulum in the HC/AD group.

**Figure 8:**
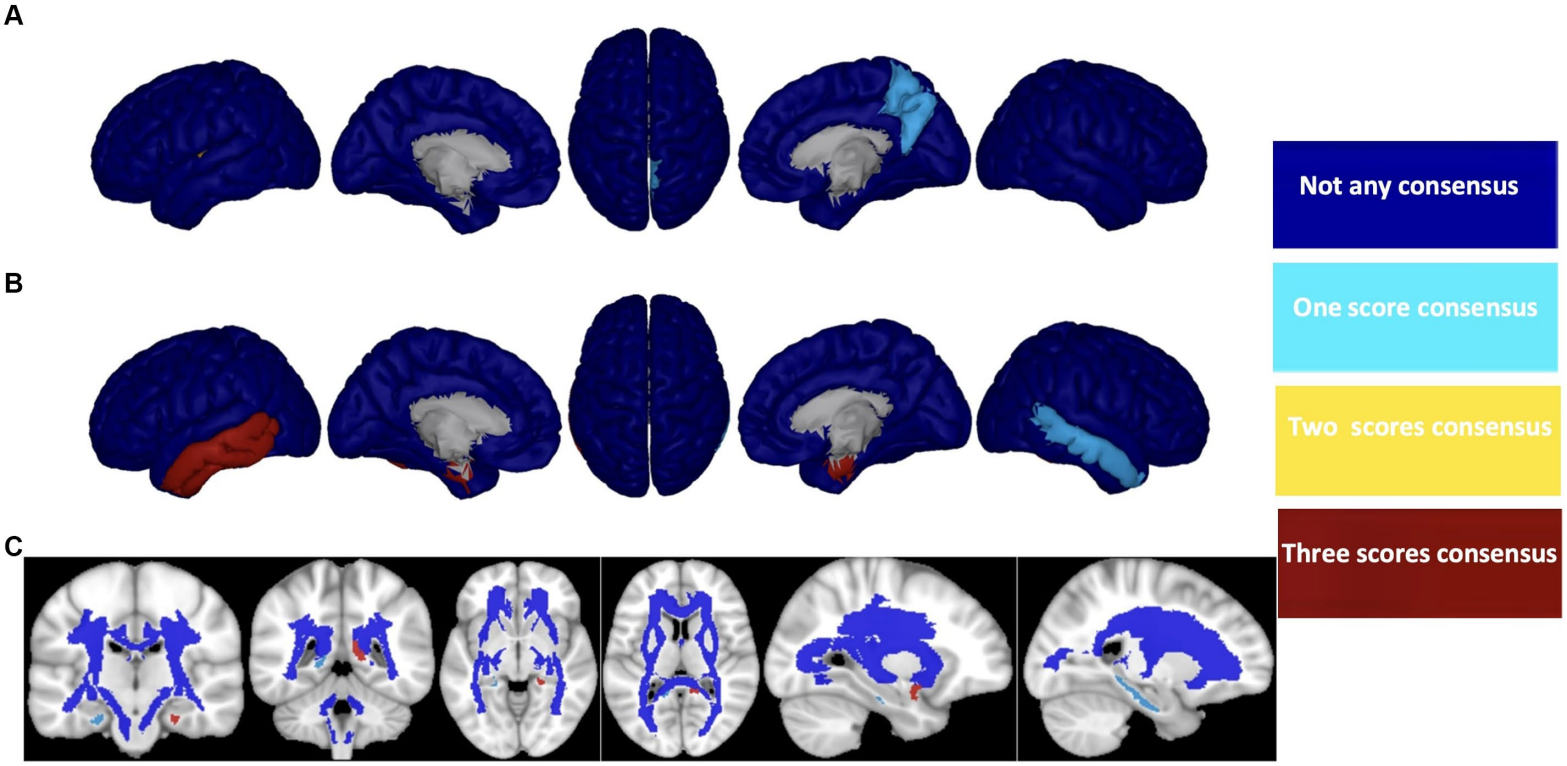
Consensus top-ranked brain regions between three cognitive tests prediction (MMSE, CDRSB, and ADAS) in HC/MCI/AD group and based on **(A)** Aβ, **(B)** ATH, and **(C)** MD. The color map is calculated based on consensus between feature importance.

After careful consideration, we chose to focus on the HC/MCI/AD group, which encompasses all three cohorts and identified the three most effective biomarkers for each neuroimaging modality (Aβ, ATH, and MD). We used these biomarkers to demonstrate a consensus on the top brain regions for predicting three cognitive scores (i.e., MMSE, CDRSB, and ADAS), as illustrated in Figure 8. For each biomarker, we identified four overlap states: no consensus, one consensus on the cognitive scores, two consensuses on cognitive scores, and consensus on all three cognitive scores. Despite differences in biomarkers, we identified six brain regions that consistently emerged as the top regions for predicting all three cognitive scores: the right transverse temporal region for Aβ, the left and right entorhinal cortex, the left inferior temporal gyrus, the left middle temporal gyrus for ATH, and the left uncinate fasciculus for MD.

## 4 Discussion

AD is a complex neurological condition that affects various brain regions and cognitive functions. To better understand the link between cognitive decline and brain changes in AD, it is crucial to identify and monitor effective biomarkers and their associated brain regions. In this study, we present a framework based on ERT to identify the brain regions that are linked to cognitive abilities. The main objectives of this study are to identify the most effective biomarkers associated with cognitive scores and to tie these biomarkers to specific brain regions. Our findings reveal that Aβ outperforms other biomarkers regarding prediction performance (MSE and correlation between predicted and actual values), but it does not appear to have a significantly different association with cognitive decline compared with other biomarkers in the absence of an AD diagnosis. The association between cognitive decline and Aβ starts at the late onset of the disease. Our second finding suggests that volumetric measures, such as ATH, are strongly associated with cognitive scores, not only in early stages (HC/MCI group) but also in late/symptomatic stages (MCI/AD group). Additionally, we found that ADAS is associated with almost all neuroimaging biomarkers in this study and in all groups of subjects, regardless of their symptomatology. Thus, ADAS appears to be a cognitive test that can track brain changes based on neuroimaging biomarkers throughout the early/asymptomatic to late/symptomatic phases of the disease. Lastly, our study demonstrates that the ERT technique can capture critical brain regions that have strong associations with all three cognitive scores throughout the early to late stages of the disease. These regions include (a) right transverse temporal (Aβ); (b) left and right entorhinal cortex, left inferior temporal gyrus and left middle temporal gyrus (ATH); and (c) left uncinate fasciculus (MD).

Previous machine learning-based AD studies have demonstrated that combining information from different neuroimaging modalities can improve classification and regression performances (Tong et al., 2017; Hojjati et al., 2019; Tabarestani et al., 2020). Our study also supports the notion that biomarkers from different neuroimaging techniques are complementary and can offer a better understanding of AD than using each biomarker or technique alone (Baron et al., 2001; Frisoni et al., 2002; Rose et al., 2006; Langbaum et al., 2009; Rathore et al., 2017). Although combining different feature domains may enhance the performance of machine learning methods, it may not fully utilize the complementary information present in each biomarker, and it remains challenging to understand the contribution of each feature in modality and link the feature to specific brain regions. Therefore, we aimed to separately utilize multiple neuroimaging biomarkers to predict cognitive scores in AD to handle the discrepancy between feature domains and gain new insights into the complex changes in the brain associated with AD.

Our results showed that Aβ PET consistently outperformed the other eight MRI-based biomarkers in predicting cognitive scores. Previous research has shown that even healthy older adults with high amyloid burden have lower cognitive performance, and high levels of Aβ are strongly related to progressive cognitive decline, particularly in episodic memory and executive function (Sperling et al., 2013; Villemagne et al., 2013; Baker et al., 2017). Longitudinal studies have shown that levels of Aβ strongly related to progressive cognitive decline, and mostly affected episodic memory and executive function (Villemagne et al., 2013; Baker et al., 2017). Despite Aβ performing well in predicting cognitive scores, the calculated NI was most significant in late/symptomatic AD stages. On the other hand, volumetric measures based on sMRI, particularly ATH, showed a relatively higher NI from early/asymptomatic to late/symptomatic AD stages in comparison with Aβ. Among DTI biomarkers, MD indicated that WM biomarker is strongly associated with cognitive scores (Lee et al., 2017). These findings suggest that exploring the differences between feature domains could provide novel insights into the intricate mechanisms of AD, rather than solely focusing on improving machine learning performance by using the best modality or integrating multimodal features.

Although each biomarker and neuroimaging technique can provide valuable insights into AD, their direct and indirect relationships with each other remain unclear, which raises questions about their individual independent contributions to cognitive decline. Moreover, the lack of clarity surrounding the simultaneous and delayed relationships between these biomarkers plays a critical role in understanding the heterogeneity of AD, which remains an unresolved challenge. This ambiguity is reflected in conflicting reports regarding the relationship between the two best biomarkers in our study: Aβ and ATH. Some studies suggest that an increase in Aβ deposition is linked with neurodegeneration, such as cortical thinning and/or lower volume (Becker et al., 2009; Glodzik et al., 2012; Doré et al., 2013; Kaffashian et al., 2015; Llado-Saz et al., 2015; Susanto et al., 2015; Hedden et al., 2016; Sala-Llonch et al., 2017; Ten Kate et al., 2018), while others report the opposite, where higher Aβ deposition is associated with cortical thickening and/or increased volume (Whitwell et al., 2013; Rahayel et al., 2019; Batzu et al., 2020; Harrison et al., 2021; Hojjati et al., 2023). Fortea et al. (2011) examined the association between Aβ values and cortical thickness in a group of cognitively preserved individuals and found a complex and nonlinear (inverted-U shaped) relationship between Aβ values and cortical thickness in various brain regions. They reported that changes in cortical thickness in regions like temporoparietal areas and precuneus were linked to intermediate Aβ values that may precede cortical thinning. Wirth et al. (2013) investigated non-Aβ factors of neurodegeneration within AD regions in older HC adults and found that many had neurodegenerative biomarker abnormalities in AD-affected brain regions, despite having normal Aβ levels. This evidence suggests that neurodegenerative patterns similar to AD can also develop through non-Aβ pathways and affect cognition in older adults without Aβ burden (Becker et al., 2011; Doherty et al., 2015).

Lastly, in order to better understand the relationship between cognitive scores and biomarkers, it is essential not only to determine the most effective biomarker for each neuroimaging modality, along with their respective associations with cognitive scores but also to identify the most significant brain regions for each biomarker and modality. The ERT framework utilized in this study generated a set of features (i.e., brain regions) that were weighted and ranked based on their predictive power for cognitive scores. Prior research findings align with our results, suggesting that cognitive decline is linked to pathologies and atrophy in the temporal lobe of the brain (Desikan et al., 2011). Specifically, critical biomarkers such as the transverse temporal gyrus and precuneus cortex have been identified in the cognitive decline associated with Aβ (Wu et al., 2016). Additionally, the cortical thickness of the entorhinal cortex has been independently and additively associated with declining memory, while different temporal regions have been identified as critical biomarkers in AD-related memory decline (Velayudhan et al., 2013; Knopman et al., 2019). Finally, our results are consistent with previous research that has demonstrated a significant negative association between cognitive scores and white matter integrity in the cingulum and the uncinate fasciculus (Li et al., 2018; Luo et al., 2020).

AD is a complex and multifaceted illness that impacts the brain and is linked to cognitive deterioration. The symptoms of AD can manifest differently, as can the underlying biological transformations in the brain. Additionally, there are several subtypes of AD that vary in the distribution of abnormal pathologies and patterns of brain changes. Employing an interdisciplinary approach to AD entails drawing on diverse neuroimaging modalities to gain a more comprehensive understanding of the disease and its underlying mechanisms. Grasping the role of each biomarker in cognitive decline is vital in overcoming the heterogeneity of AD and developing more effective treatments.

Recognizing and addressing specific potential limitations in this study is crucial for guiding future research efforts. Firstly, our attempt to maximize subject inclusion for each modality of imaging resulted in varying numbers of features (brain regions) and subjects across different imaging modalities. In other words, subjects did not necessarily undergo all three neuroimaging modalities, potentially impacting analyses due to the heterogeneity of AD and the possibility that subjects used in different prediction tasks may be in different disease stages. To enhance the robustness of our findings, it is essential to validate results in independent datasets with the same sample represented in all modalities. Another limitation concerns the sample size of the DTI modality, which was smaller compared to other modalities. This discrepancy may influence the prediction task and result in lower performance for this modality. Additionally, the absence of other robust biomarkers such as tau and FDG in cognitive scores is a noteworthy limitation. However, the strong associations between these biomarkers and cognitive scores have been well-documented by previous studies. Despite these limitations, they should be viewed as opportunities for further research to build upon and refine our understanding.

## 5 Conclusion

In this study, we investigated the potential of an interdisciplinary approach to predict cognitive scores in AD by utilizing multimodal neuroimaging biomarkers. Our proposed ERT prediction model achieved this goal by identifying the most associated biomarkers, especially in the early stages of the disease, and mapping their importance to specific brain regions. Our findings revealed that Aβ, ATH, and MD biomarkers, derived from PET, sMRI, and DTI, respectively, were strongly associated with cognitive scores in AD. Among the nine biomarkers examined, ATH had the strongest association with the cognitive disorder in the HC/MCI group (early stage of AD), while Aβ biomarkers were most effective in predicting cognitive scores in AD-stage subjects. Furthermore, we found that ADAS decline was best explained by almost all considered biomarkers, unlike MMSE and CDRSB. Our results showed that cognitive decline was primarily driven by the right transverse temporal gyrus (based on Aβ), left and right entorhinal cortex, left inferior temporal gyrus, left middle temporal gyrus, and left uncinate fasciculus. These findings highlight the importance of an interdisciplinary approach to understanding the underlying mechanisms of AD and may help in the development of more effective treatments.

## Data availability statement

Publicly available datasets were analyzed in this study. This data can be found here: adni.loni.usc.edu.

## Ethics statement

As per Alzheimer’s Disease Neuroimaging Initiative (ADNI) protocols, all procedures conducted in studies involving human participants adhered to ethical standards. The ADNI data collection was conducted following written informed consent from the participants. Further details can be found at adni.loni.usc.edu.

## Author contributions

SH: Conceptualization, Data curation, Formal analysis, Methodology, Software, Validation, Visualization, Writing – original draft, Writing – review & editing. AB-F: Conceptualization, Investigation, Methodology, Resources, Supervision, Writing – review & editing, Writing – original draft.

## Footnotes

^1^ https://tadpole.grand-challenge.org

^2^Data used in this article were originally obtained from the Alzheimer’s Disease Neuroimaging Initiative (ADNI) database (adni.loni.usc.edu). The ADNI was launched in 2003 as a public-private partnership, led by Principal Investigator Michael W. Weiner, MD. The primary goal of ADNI has been to test whether serial magnetic resonance imaging (MRI), positron emission tomography (PET), other biological markers, and clinical and neuropsychological assessment can be combined to measure the progression of mild cognitive impairment (MCI) and early Alzheimer’s disease (AD).
