# Supplementary material for: Seeing beyond the symptoms: biomarkers and brain regions linked to cognitive decline in Alzheimer’s disease
Source: Front Aging Neurosci. 2024 May 15;16:1356656. doi: 10.3389/fnagi.2024.1356656 (PMC11135344; doi:10.3389/fnagi.2024.1356656)
Supplement: Supplementary file 1 [file Data_Sheet_1.docx]

**Supplementary materials:**

Table S1. List of Freesurfer brain regions used as features for our prediction approach in three neuroimaging modalities and across all nine biomarkers. The cortical regions in the Desikan-Killiany atlas are highlighted in red font.

| SMRI | | | DTI | PET |
| --- | --- | --- | --- | --- |
| VGM and ATH | **SA** | **VWM** | **FA, MD, RD, and LD** | **Aβ** |
| ctx-lh-bankssts | ctx-lh-bankssts | RightPallidum | Corticospinal tract right | ctx-lh-bankssts |
| ctx-lh-caudalmiddlefrontal | ctx-lh-caudalmiddlefrontal | RightPutamen | Inferior cerebellar peduncle left | ctx-lh-caudalmiddlefrontal |
| ctx-lh-cuneus | ctx-lh-cuneus | LeftAccumbensArea | Inferior cerebellar peduncle right | ctx-lh-cuneus |
| ctx-lh-entorhinal | ctx-lh-entorhinal | RightThalamus | Medial lemniscus left | ctx-lh-entorhinal |
| ctx-lh-fusiform | ctx-lh-fusiform | RightUndetermined | Medial lemniscus right | ctx-lh-fusiform |
| ctx-lh-inferiorparietal | ctx-lh-inferiorparietal | RightVentralDC | Superior cerebellar peduncle left | ctx-lh-inferiorparietal |
| ctx-lh-inferiortemporal | ctx-lh-inferiortemporal | RightVessel | Superior cerebellar peduncle right | ctx-lh-inferiortemporal |
| ctx-lh-isthmuscingulate | ctx-lh-isthmuscingulate | RightWMHypoIntensities | Cerebral peduncle left | ctx-lh-isthmuscingulate |
| ctx-lh-lateraloccipital | ctx-lh-lateraloccipital | ThirdVentricle | Cerebral peduncle right | ctx-lh-lateraloccipital |
| ctx-lh-lateralorbitofrontal | ctx-lh-lateralorbitofrontal | LeftAmygdala | Anterior limb of internal capsule left | ctx-lh-lateralorbitofrontal |
| ctx-lh-lingual | ctx-lh-lingual | LeftCaudate | Anterior limb of internal capsule right | ctx-lh-lingual |
| ctx-lh-medialorbitofrontal | ctx-lh-medialorbitofrontal | LeftCerebellumCortex | Posterior limb of internal capsule left | ctx-lh-medialorbitofrontal |
| ctx-lh-middletemporal | ctx-lh-middletemporal | LeftCerebellumWM | Posterior limb of internal capsule right | ctx-lh-middletemporal |
| ctx-lh-parahippocampal | ctx-lh-parahippocampal | LeftCerebralCortex | Posterior thalamic radiation left | ctx-lh-parahippocampal |
| ctx-lh-paracentral | ctx-lh-paracentral | LeftChoroidPlexus | Posterior thalamic radiation right | ctx-lh-paracentral |
| ctx-lh-parsopercularis | ctx-lh-parsopercularis | LeftHippocampus | Anterior corona radiata left | ctx-lh-parsopercularis |
| ctx-lh-parsorbitalis | ctx-lh-parsorbitalis | CorpusCallosumAnterior | Anterior corona radiata right | ctx-lh-parsorbitalis |
| ctx-lh-parstriangularis | ctx-lh-parstriangularis | LeftInferiorLateralVentricle | Superior corona radiata left | ctx-lh-parstriangularis |
| ctx-lh-pericalcarine | ctx-lh-pericalcarine | LeftLateralVentricle | Superior corona radiata right | ctx-lh-pericalcarine |
| ctx-lh-postcentral | ctx-lh-postcentral | CorpusCallosumCentral | Posterior corona radiata left | ctx-lh-postcentral |
| ctx-lh-posteriorcingulate | ctx-lh-posteriorcingulate | LeftNonWMHypoIntensities | Posterior corona radiata right | ctx-lh-posteriorcingulate |
| ctx-lh-precentral | ctx-lh-precentral | LeftPallidum | Cingulum left | ctx-lh-precentral |
| ctx-lh-precuneus | ctx-lh-precuneus | CorpusCallosumMidAnterior | Cingulum right | ctx-lh-precuneus |
| ctx-lh-rostralmiddlefrontal | ctx-lh-rostralmiddlefrontal | LeftPutamen | Cingulum (hippocampus) left | ctx-lh-rostralmiddlefrontal |
| ctx-lh-superiorfrontal | ctx-lh-superiorfrontal | CorpusCallosumMidPosterior | Cingulum (hippocampus) right | ctx-lh-superiorfrontal |
| ctx-lh-superiorparietal | ctx-lh-superiorparietal | LeftThalamus | Fornix (cres) / Stria terminalis left | ctx-lh-superiorparietal |
| ctx-lh-superiortemporal | ctx-lh-superiortemporal | LeftUndetermined | Fornix (cres) / Stria terminalis right | ctx-lh-superiortemporal |
| ctx-lh-supramarginal | ctx-lh-supramarginal | LeftVentralDC | Superior longitudinal fasciculus left | ctx-lh-supramarginal |
| ctx-lh-frontalpole | ctx-lh-frontalpole | LeftVessel | Superior longitudinal fasciculus right | ctx-lh-frontalpole |
| ctx-lh-temporalpole | ctx-lh-temporalpole | OpticChiasm | Superior fronto-occipital fasciculus left | ctx-lh-temporalpole |
| ctx-lh-transversetemporal | ctx-lh-transversetemporal | CorpusCallosumPosterior | Superior fronto-occipital fasciculus right | ctx-lh-transversetemporal |
| ctx-lh-insula | ctx-lh-insula | RightAccumbensArea | Inferior fronto-occipital fasciculus left | ctx-lh-insula |
| ctx-lh-cuadalanteriorcingulate | ctx-lh-cuadalanteriorcingulate | RightAmygdala | Inferior fronto-occipital fasciculus right | ctx-lh-cuadalanteriorcingulate |
| ctx-lh-rostralanteriorcingulate | ctx-lh-rostralanteriorcingulate | RightCaudate | Sagittal stratum left | ctx-lh-rostralanteriorcingulate |
| ctx-rh-bankssts | ctx-rh-bankssts | RightCerebellumCortex | Sagittal stratum right | ctx-rh-bankssts |
| ctx-rh-caudalmiddlefrontal | ctx-rh-caudalmiddlefrontal | RightCerebellumWM | External capsule left | ctx-rh-caudalmiddlefrontal |
| ctx-rh-cuneus | ctx-rh-cuneus | RightCerebralCortex | External capsule right | ctx-rh-cuneus |
| ctx-rh-entorhinal | ctx-rh-entorhinal | RightCerebralWM | Uncinate fasciculus left | ctx-rh-entorhinal |
| ctx-rh-fusiform | ctx-rh-fusiform | RightChoroidPlexus | Uncinate fasciculus right | ctx-rh-fusiform |
| ctx-rh-inferiorparietal | ctx-rh-inferiorparietal | RightHippocampus | Fornix left | ctx-rh-inferiorparietal |
| ctx-rh-inferiortemporal | ctx-rh-inferiortemporal | RightInferiorLateralVentricle | Fornix right | ctx-rh-inferiortemporal |
| ctx-rh-isthmuscingulate | ctx-rh-isthmuscingulate | FifthVentricle | Genu of corpus callosum left | ctx-rh-isthmuscingulate |
| ctx-rh-lateraloccipital | ctx-rh-lateraloccipital | RightInterior | Genu of corpus callosum right | ctx-rh-lateraloccipital |
| ctx-rh-lateralorbitofrontal | ctx-rh-lateralorbitofrontal | RightLateralVentricle | Body of corpus callosum left | ctx-rh-lateralorbitofrontal |
| ctx-rh-lingual | ctx-rh-lingual | FourthVentricle | Body of corpus callosum right | ctx-rh-lingual |
| ctx-rh-medialorbitofrontal | ctx-rh-medialorbitofrontal |  | Splenium of corpus callosum left | ctx-rh-medialorbitofrontal |
| ctx-rh-middletemporal | ctx-rh-middletemporal |  | Splenium of corpus callosum right | ctx-rh-middletemporal |
| ctx-rh-parahippocampal | ctx-rh-parahippocampal |  | Retrolenticular part of internal capsule left | ctx-rh-parahippocampal |
| ctx-rh-paracentral | ctx-rh-paracentral |  | Retrolenticular part of internal capsule right | ctx-rh-paracentral |
| ctx-rh-parsopercularis | ctx-rh-parsopercularis |  | Tapatum left | ctx-rh-parsopercularis |
| ctx-rh-parsorbitalis | ctx-rh-parsorbitalis |  | Tapatum right | ctx-rh-parsorbitalis |
| ctx-rh-parstriangularis | ctx-rh-parstriangularis |  | Bilateral genu of the corpus callosum | ctx-rh-parstriangularis |
| ctx-rh-pericalcarine | ctx-rh-pericalcarine |  | Bilateral body of the corpus callosum | ctx-rh-pericalcarine |
| ctx-rh-postcentral | ctx-rh-postcentral |  | Bilateral splenium of the corpus callosum | ctx-rh-postcentral |
| ctx-rh-posteriorcingulate | ctx-rh-posteriorcingulate |  | Bilateral full corpus callosum | ctx-rh-posteriorcingulate |
| ctx-rh-precentral | ctx-rh-precentral |  | Bilateral fornix | ctx-rh-precentral |
| ctx-rh-precuneus | ctx-rh-precuneus |  | Corticospinal tract left | ctx-rh-precuneus |
| ctx-rh-rostralmiddlefrontal | ctx-rh-rostralmiddlefrontal |  |  | ctx-rh-rostralmiddlefrontal |
| ctx-rh-superiorfrontal | ctx-rh-superiorfrontal |  |  | ctx-rh-superiorfrontal |
| ctx-rh-superiorparietal | ctx-rh-superiorparietal |  |  | ctx-rh-superiorparietal |
| ctx-rh-superiortemporal | ctx-rh-superiortemporal |  |  | ctx-rh-superiortemporal |
| ctx-rh-supramarginal | ctx-rh-supramarginal |  |  | ctx-rh-supramarginal |
| ctx-rh-frontalpole | ctx-rh-frontalpole |  |  | ctx-rh-frontalpole |
| ctx-rh-temporalpole | ctx-rh-temporalpole |  |  | ctx-rh-temporalpole |
| ctx-rh-transversetemporal | ctx-rh-transversetemporal |  |  | ctx-rh-transversetemporal |
| ctx-rh-insula | ctx-rh-insula |  |  | ctx-rh-insula |
| ctx-rh-cuadalanteriorcingulate | ctx-rh-cuadalanteriorcingulate |  |  | ctx-rh-cuadalanteriorcingulate |
| ctx-rh-rostralanteriorcingulate | ctx-rh-rostralanteriorcingulate |  |  | ctx-rh-rostralanteriorcingulate |
|  | ctx-lh-subcallosal |  |  | 3rd-ventricle |
|  | ctx-rh-subcallosal |  |  | 4th-ventricle |
|  |  |  |  | cc-anterior |
|  |  |  |  | cc-central |
|  |  |  |  | cc-mid-anterior |
|  |  |  |  | cc-mid-posterior |
|  |  |  |  | cc-posterior |
|  |  |  |  | left- subcallosal |
|  |  |  |  | left-accumbens-area |
|  |  |  |  | left-amygdala |
|  |  |  |  | left-caudate |
|  |  |  |  | left-cerebellum-cortex |
|  |  |  |  | left-cerebellum-white-matter |
|  |  |  |  | left-cerebral-white-matter |
|  |  |  |  | left-choroid-plexus |
|  |  |  |  | left-hippocampus |
|  |  |  |  | left-inf-lat-vent |
|  |  |  |  | left-lateral-ventricle |
|  |  |  |  | left-pallidum |
|  |  |  |  | left-putamen |
|  |  |  |  | left-thalamus-proper |
|  |  |  |  | left-ventraldc |
|  |  |  |  | left-vessel |
|  |  |  |  | non-wm-hypointensities |
|  |  |  |  | optic-chiasm |
|  |  |  |  | right-accumbens-area |
|  |  |  |  | right-amygdala |
|  |  |  |  | right-caudate |
|  |  |  |  | right-cerebellum-cortex |
|  |  |  |  | right-cerebellum-white-matter |
|  |  |  |  | right-cerebral-white-matter |
|  |  |  |  | right-choroid-plexus |
|  |  |  |  | right-hippocampus |
|  |  |  |  | right-inf-lat-vent |
|  |  |  |  | right-lateral-ventricle |
|  |  |  |  | right-pallidum |
|  |  |  |  | right-putamen |
|  |  |  |  | right-thalamus-proper |
|  |  |  |  | right-ventraldc |
|  |  |  |  | right-vessel |
|  |  |  |  | right- subcallosal |


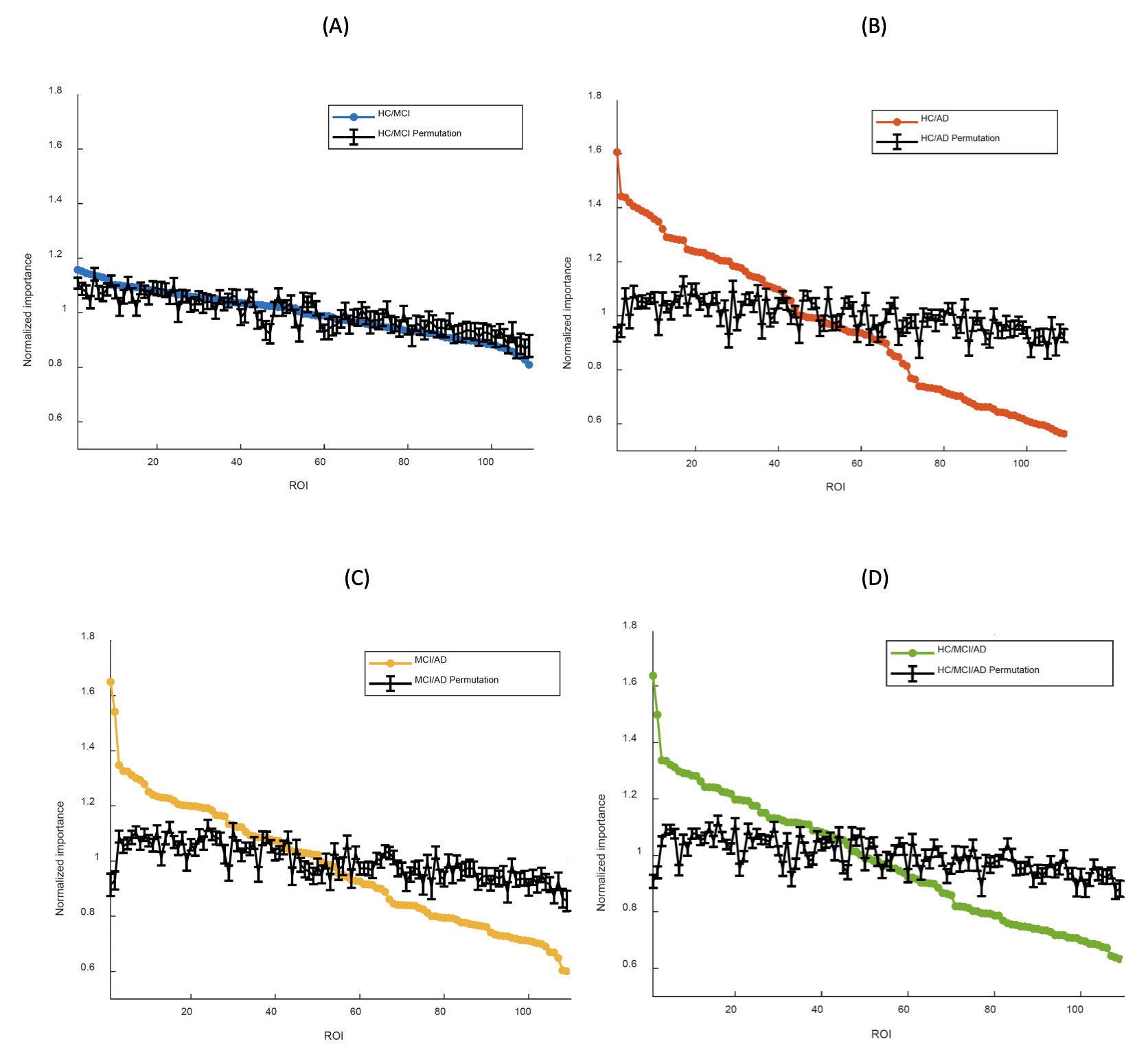


Figure S1. Normalized feature importance extrcated from ERT models for predicting MMSE scores using Aβ in four different combinations of subject groups: (A) HC/MCI, (B) HC/AD, (C) MCI/AD, and (D) HC/MCI/AD. The x-axis represents 109 cortical and subcortical brain regions used in the prediction of the MMSE score based on Aβ. The y-axis represents the normalized importance value in two distinct conditions: one with randomly permuted values (depicted in black) and the other with actual cognitive measures. Larger values of normalized importance in brain regions indicate a higher association between that region and the MMSE score.


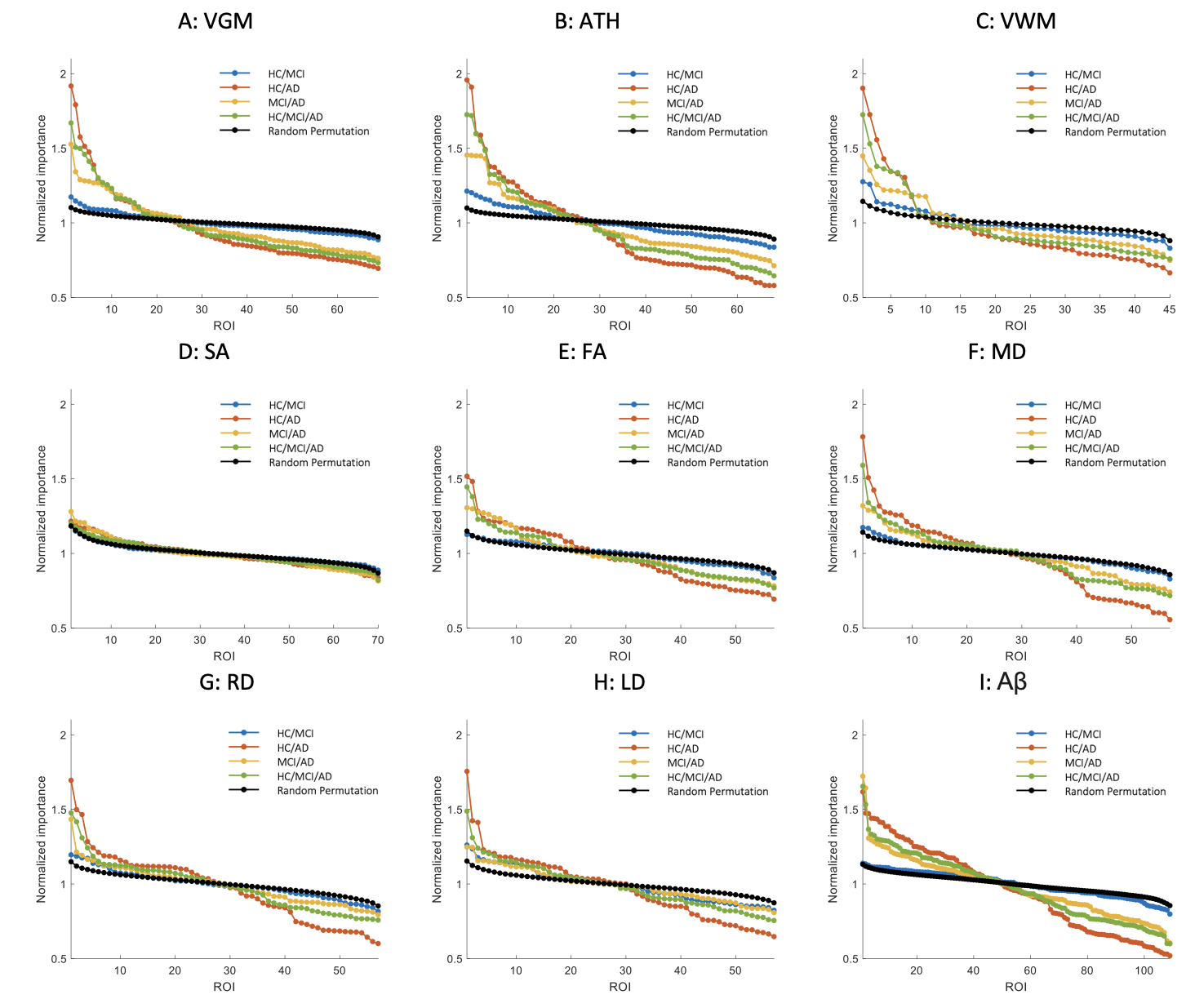


Figure S2. Feature importance for the prediction of CDRSB score based on nine different biomarkers: A) VGM, B) ATH, C) VWM, D) SA, E) FA, F) MD, G) RD, H) LD, and I) Aβ. The x-axis represents the brain regions considered in the prediction based on each biomarker. The y-axis represents the normalized importance value for four different combinations of groups and a random permutation. Larger values of normalized importance in brain regions show a higher association between that region's feature and the CDRSB score.

**
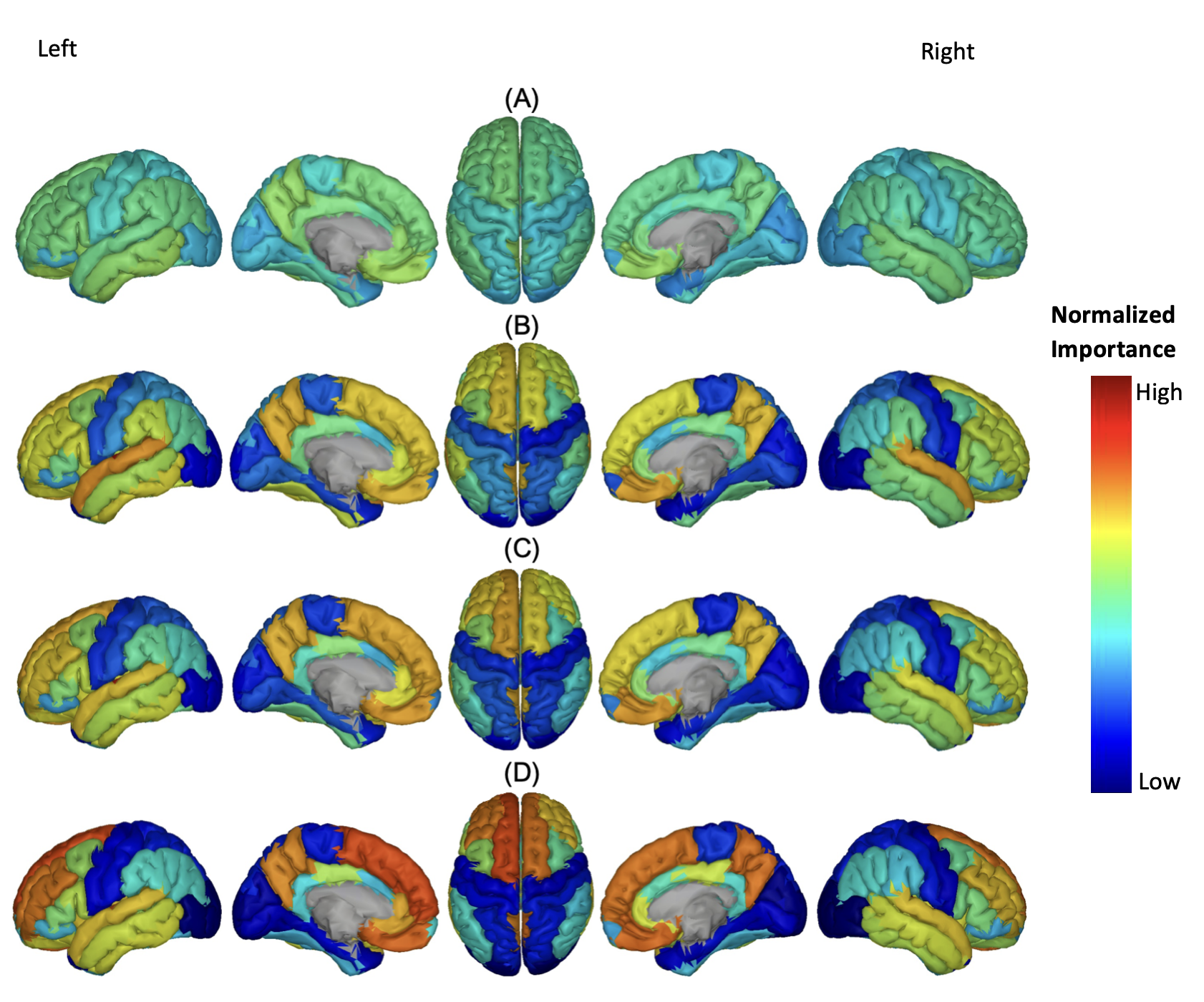
**

Figure S3. Feature importance of Aβ of the cortical brain regions for predicting the MMSE score in four different combinations of groups: (A) HC/MCI, (B) MCI/AD, (C) HC/MCI/AD, and (D) HC/AD. The groups of subjects from top to bottom show a gradual increase in cognitive decline from normal aging to AD. The color map was calculated based on normalized feature importance values, which indicate the degree of association between each cortical brain region's feature and the MMSE score.


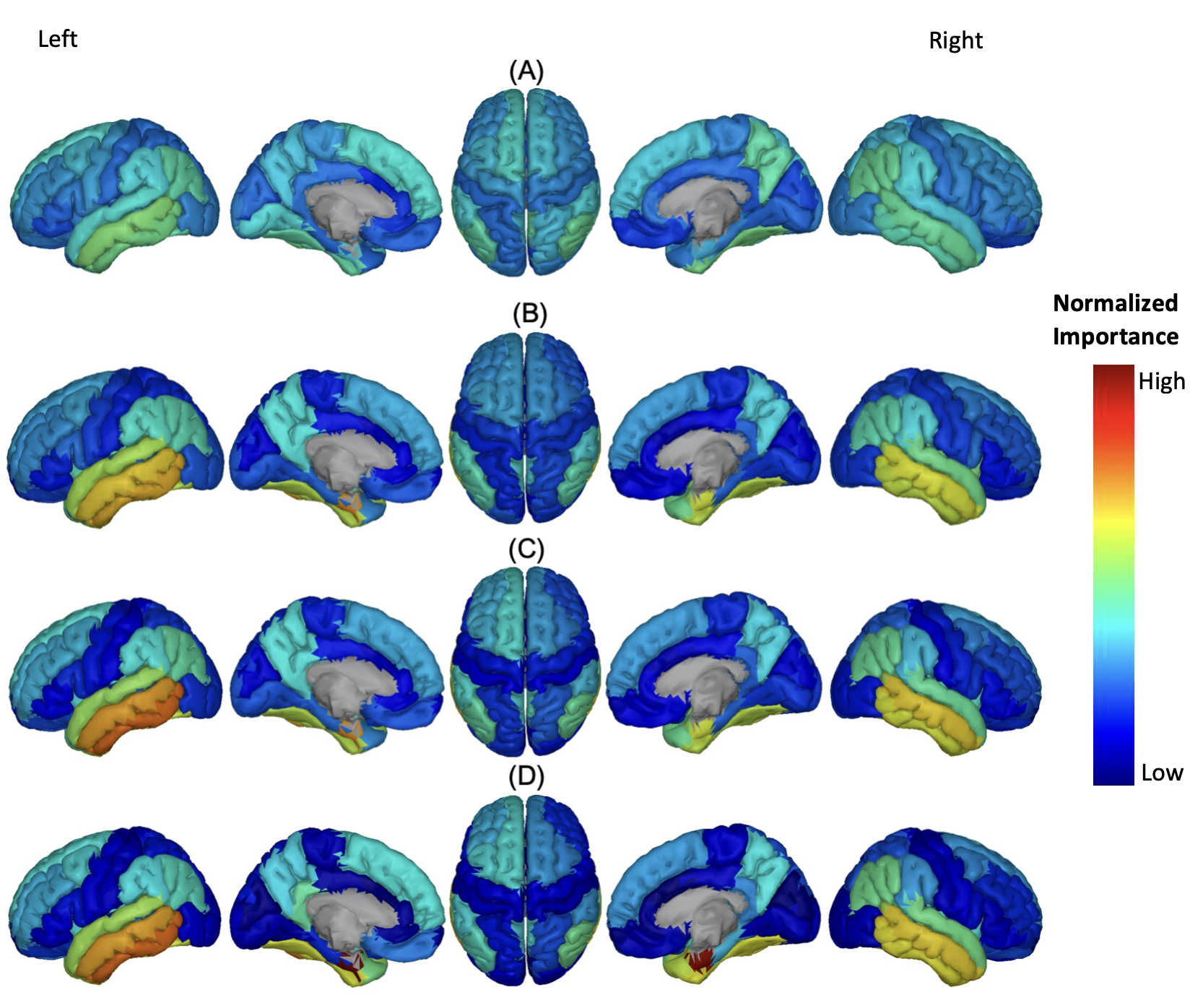
Figure S4. Feature importance of ATH of the cortical brain regions for the prediction of MMSE score in four combinations of groups: (A) HC/MCI, (B) MCI/AD, (C) HC/MCI/AD, and (D) HC/AD. From top to bottom, groups of subjects have small to large differences in the cognitive scores from normal aging to AD. The color map was calculated based on normalized feature importance values.

**
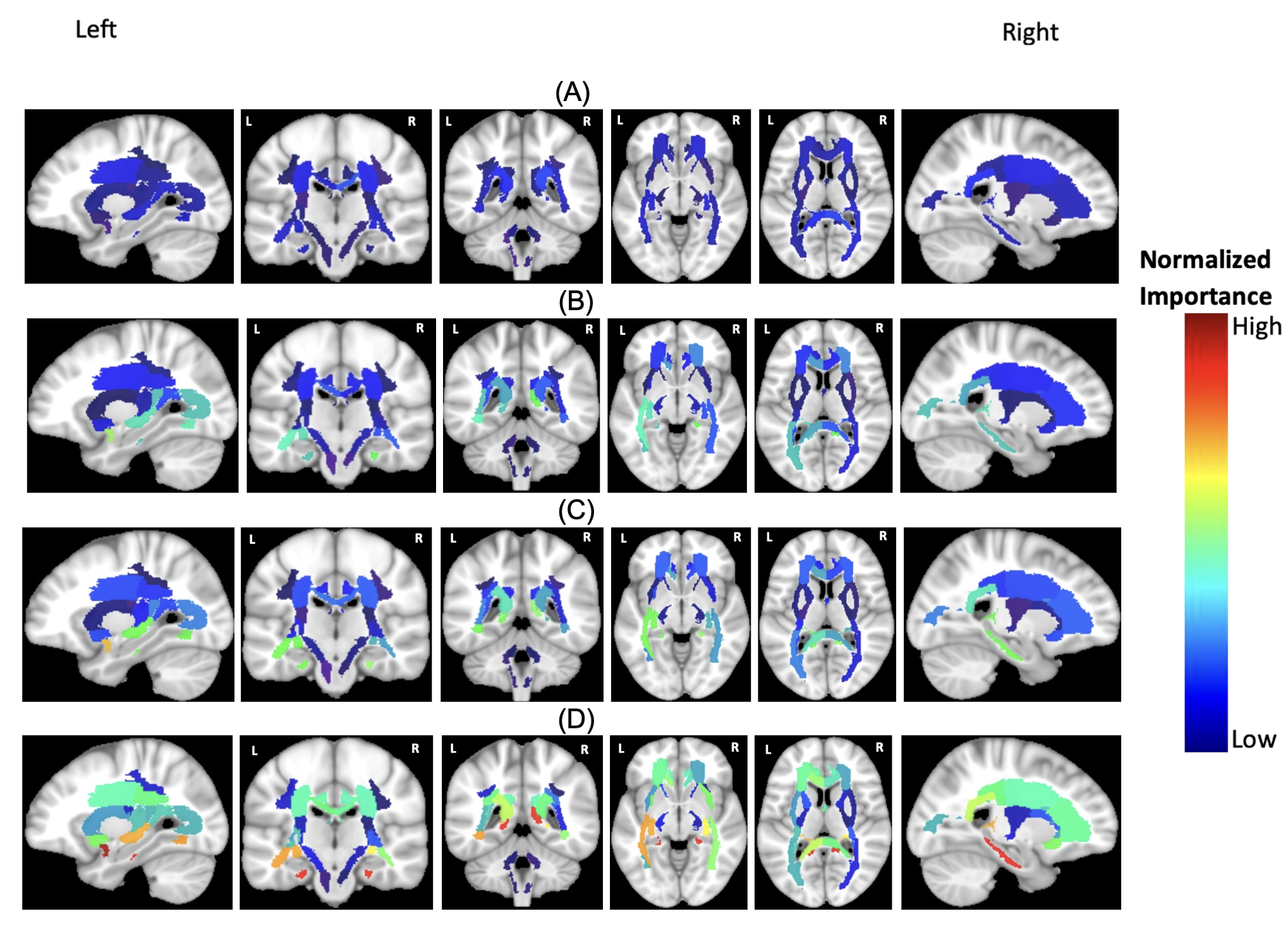
**

Figure S5. Feature importance of MD of the cortical brain regions for the prediction of MMSE score in four combinations of groups: (A) HC/MCI, (B) MCI/AD, (C) HC/MCI/AD, and (D) HC/AD. From top to bottom, groups of subjects have small to large differences in the cognitive scores from normal aging to AD. The color map was calculated based on normalized feature importance values.


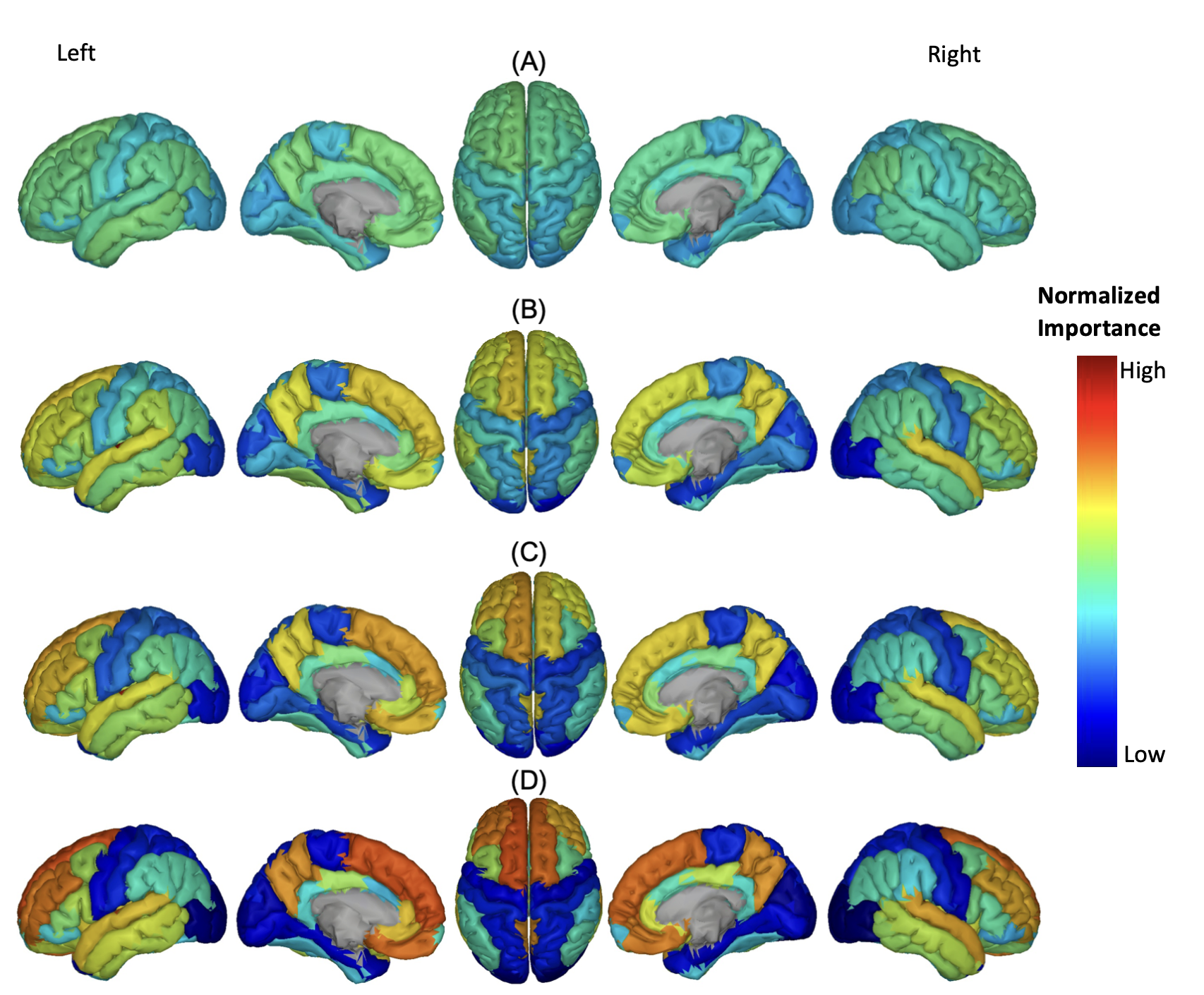


Figure S6. Feature importance of Aβ of the cortical brain regions for the prediction of CDRSB score in four combinations of groups: (A) HC/MCI, (B) MCI/AD, (C) HC/MCI/AD, and (D) HC/AD. From top to bottom, groups of subjects have small to large differences in the cognitive scores from normal aging to AD. The color map was calculated based on normalized feature importance values.


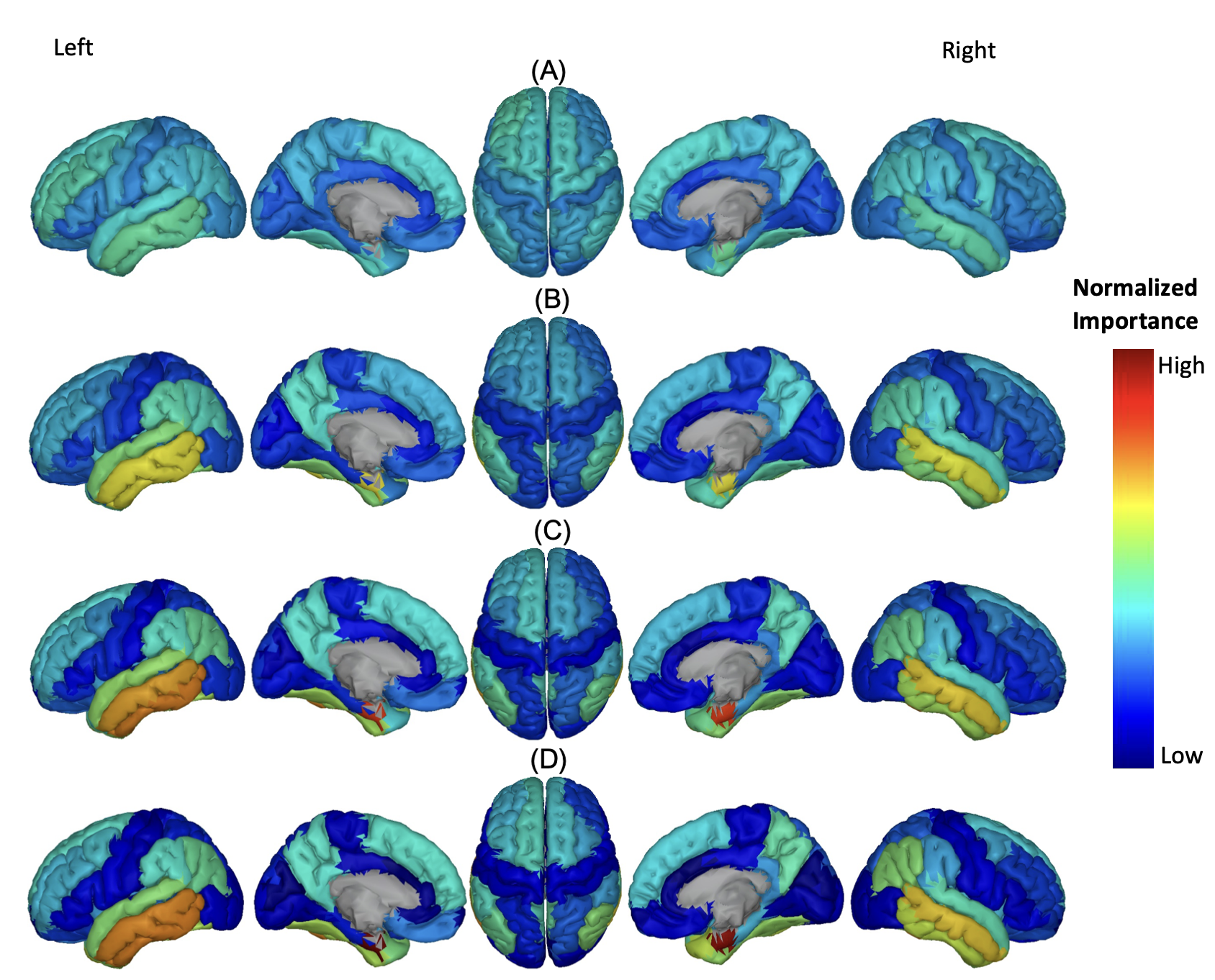


Figure S7. Feature importance of ATH of the cortical brain regions for the prediction of CDRSB score in four combinations of groups: (A) HC/MCI, (B) MCI/AD, (C) HC/MCI/AD, and (D) HC/AD. From top to bottom, groups of subjects have small to large differences in the cognitive scores from normal aging to AD. The color map was calculated based on normalized feature importance values.


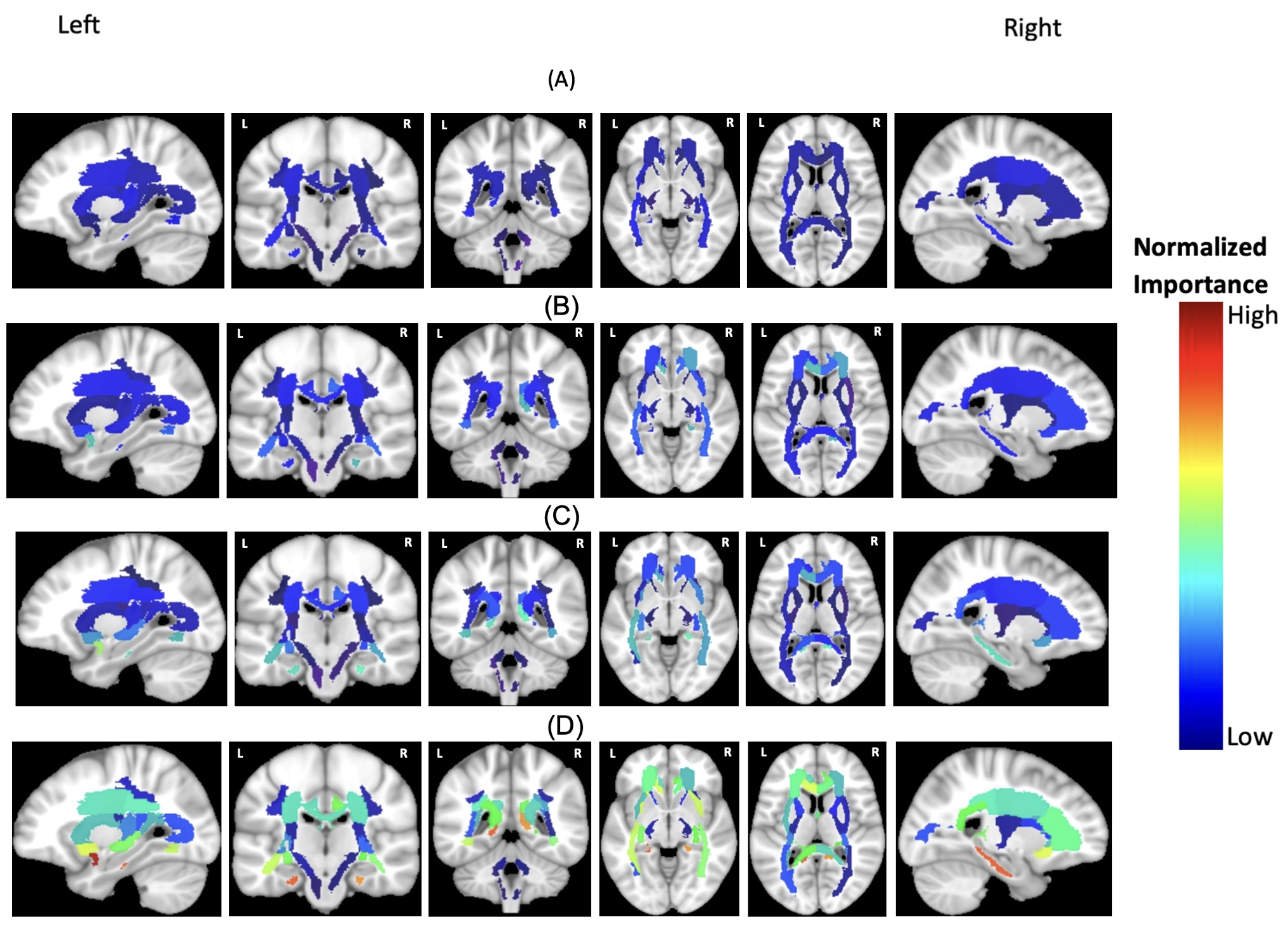
Figure S8. Feature importance of MD of the cortical brain regions for the prediction of CDRSB score in four combinations of groups: (A) HC/MCI, (B) MCI/AD, (C) HC/MCI/AD, and (D) HC/AD. From top to bottom, groups of subjects have small to large differences in the cognitive scores from normal aging to AD. The color map was calculated based on normalized feature importance values.
